# Interlayer Ions Control Spin Canting in Low-Dimensional Manganese Trimers in 12R-Ba_4_*M*Mn_3_O_12_ (*M* = Ce, Pr) Layered Perovskites

**DOI:** 10.1021/acs.inorgchem.4c03915

**Published:** 2024-12-07

**Authors:** Corlyn E. Regier, Shaun O’Donnell, Anuj Goyal, Michael J. Dzara, James Eujin Park, Robert T. Bell, Morgan J. Kramer, Joseph A. M. Paddison, Sarah Shulda, David S. Ginley, Danielle R. Yahne, Stephan Lany, Rebecca W. Smaha, Ryan A. Klein

**Affiliations:** † Department of Chemistry, Colorado State University, Fort Collins, Colorado 80523, United States; ‡ Materials, Chemical, and Computational Sciences Directorate, National Renewable Energy Laboratory, Golden, Colorado 80401, United States; § Department of Materials Science and Metallurgical Engineering, IIT Hyderabad, Kandi, Sangareddy, Telangana 502284, India; ∥ Sandia National Laboratories, P.O. Box 5800, Albuquerque, New Mexico 87185, United States; ⊥ Neutron Scattering Division, Oak Ridge National Laboratory, Oak Ridge, Tennessee 37831, United States; # Center for Neutron Research, National Institute of Standards and Technology, Gaithersburg, Maryland 20899, United States; ∇ Department of Chemical and Biomolecular Engineering, University of Delaware, Newark, Delaware 19716, United States

## Abstract

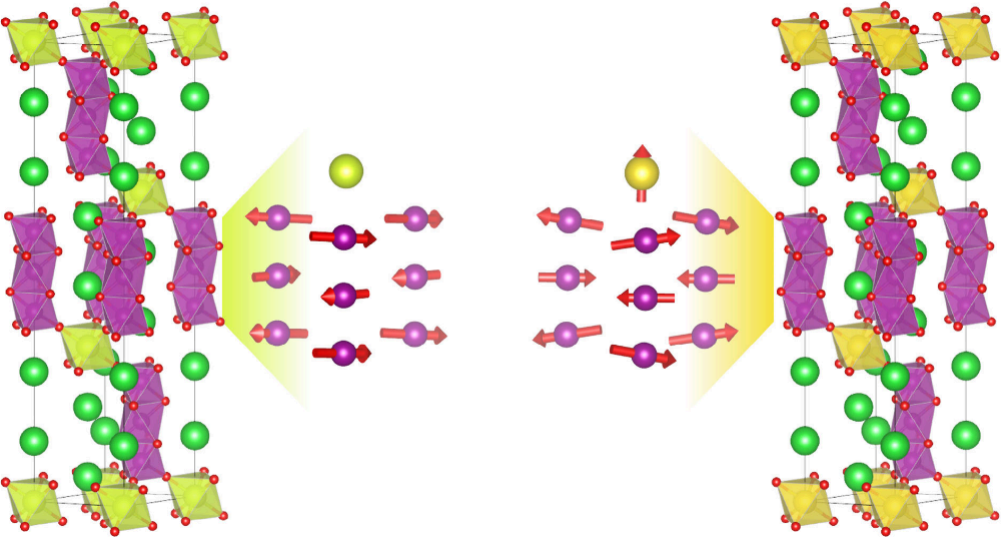

To synthetically target a specific material with select performance, the underlying relationship between structure and function must be understood. For targeting magnetic properties, such understanding is underdeveloped for a relatively new class of layered hexagonal perovskites, the 12R-Ba_4_*M*Mn_3_O_12_ family. Here, we perform a detailed magnetostructural study of the layered hexagonal perovskite materials 12R-Ba_4_*M*Mn_3_O_12_, where *M* = diamagnetic Ce^4+^ or paramagnetic *J*_eff_ ≈ 1/2 Pr^4+^. The material with *M* = Ce^4+^ is an antiferromagnet below *T*_N_ ≈ 7.75 K, while the material with *M* = Pr^4+^ exhibits more complex behavior, with a net moment below 200 K and a sharp peak in the susceptibility at *T*_N_ ≈ 12.15 K. Guided by the susceptibility data, we conduct variable-temperature powder neutron diffraction measurements to determine the magnetic structure of these two materials. The introduction of a magnetic interlayer cation cants the spins in the Mn_3_O_12_ trimers out of plane. We further characterize the crystal and electronic structures in these compounds using powder X-ray diffraction and X-ray absorption spectroscopy measurements coupled with first-principles theoretical calculations. The resulting detailed picture of the magnetic, crystal, and electronic structure will be useful for understanding the magnetism in similar 12R hexagonal perovskites and related materials.

## Abstract 2

Powder neutron diffraction measurements reveal the magnetic structures for two 12R hexagonal perovskites. The inclusion of a paramagnetic ion between the layers of otherwise isolated Mn_3_O_12_ trimers leads to spin canting.

## Introduction

Targeted functional materials design is critically important to the discovery and development of new materials with applications as permanent magnets,^1−3^ spintronic devices,^4,5^ or superconductors.^6−9^ Such informed materials design and synthesis stems from an understanding of structure–function relationships.^10−12^ One class of materials displaying fascinating magnetic and superconducting properties is the transition metal oxide perovskites.^6,9,11^ These *AB*O_3_ perovskites have garnered intense scientific interest over the past six decades for their compositional and structural tunability, which produces diverse and complex phenomena like high-*T*_C_ superconductivity and frustrated magnetism.^6−9,13−16^ These materials are extremely tunable—not only can the *A* and *B* cations be selected to achieve desired functionalities, the stoichiometry and therefore the structure type can be tuned to realize platforms with different structural motifs (and consequently bulk properties and ground states) of magnetic ions.^17−19^

**Figure 1:**
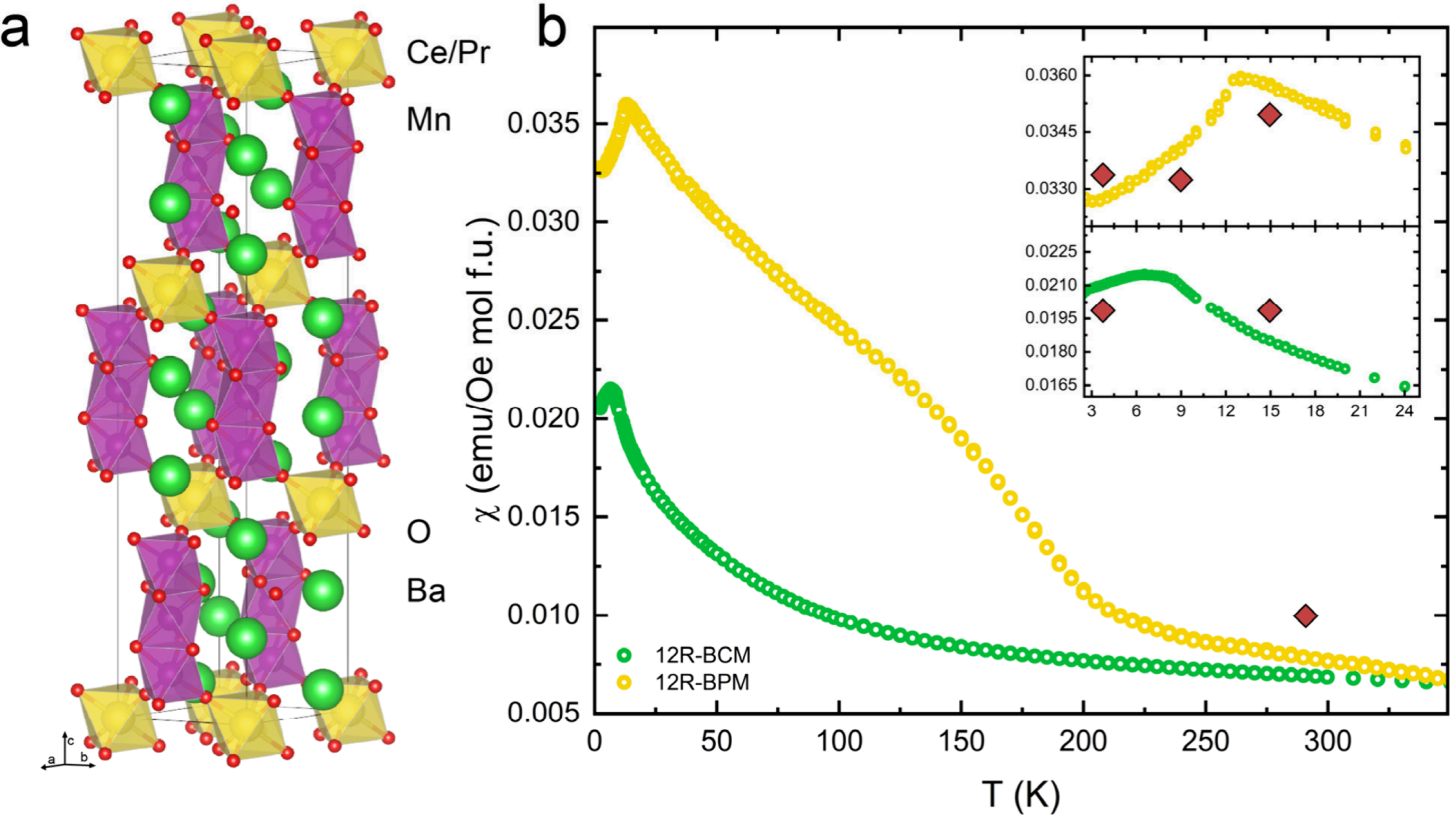
(a) The crystal structure of 12R-Ba_4_*M*Mn_3_O_12_ (*M* = Ce^4+^, Pr^4+^) contains face-sharing Mn_3_O_12_ trimers that are bridged in the crystallographic *c*-axis direction by either diamagnetic CeO_6_ octahedra or by *J*_eff_ = 1/2 PrO_6_ octahedra. Green and red spheres depict Ba and O atoms, respectively, while purple and gold polyhedra represent the coordination environments for Mn and either Ce or Pr, respectively. (b) A plot of the zero-field cooled DC magnetic susceptibility data collected at μ_0_*H* = 1 T applied field for both compounds. Long range AFM transitions are indicated by sharp peaks at *T*_N_ ≈ 7.75 K for 12R-BCM and *T*_2_ ≈ 12.15 for 12R-BPM. Magnetic data reproduced from ref (30). The inset shows the low temperature region. Maroon diamonds indicate approximate conditions where powder neutron diffraction patterns were collected to probe the crystal and magnetic structures.

To this end, a relatively new structural family of hexagonal layered perovskite oxide structures has been investigated for their magnetic properties: the 12R-Ba*M*Mn_3_O_12_ systems.^16,19^ These materials contain low-dimensional trimers of face-sharing {MnO_6_} octahedra (reminiscent of Ba_4_Mn_3_O_10_ and Ba_4_Ru_3_O_10_)^20,21^ with short metal–metal distances. The trimers are effectively magnetically isolated from each other by the Ba cations (Figure 1, left). The Mn_3_O_12_ trimers are capped in the crystallographic *c*-axis direction by *M*O_6_ octahedra that can be diamagnetic or paramagnetic. The tremendous synthetic tuneability of these materials enables fine control over the electronics at the transition ion trimers by substitution at the Ba, *M*, and Mn sites. For example, systems have been realized with varying oxidation states of the *M* site cation with *M*^3+^, *M*^4+^, and *M*^5+^, as in 12R-Ba_4_SbMn_3_O_12_,^22,23^ 12R-Ba_4_CeMn_3_O_12_,^24^ 12R-Ba_4_NbMn_3_O_12_,^25−27^ and 12R-Ba_4_TaMn_3_O_12_,^26^. The electronics can be further tuned by doping the system with *M* site cations of different oxidation states,^22^ by introducing O vacancies, and by employing a mixed metal approach at the transition ion site.^28^ These approaches all tune the number of electron spins in the transition metal trimers, controlling the bulk magnetism and allowing for the realization of a huge diversity of magnetic ground states, from diamagnets to ferromagnets, ferrimagnets, and frustrated and classical antiferromagnets (AFMs).^16,29^ In addition, cluster magnetism within the Mn_3_O_12_ trimers has been proposed in several members of this family (including 12R-Ba_4_NbMn_3_O_12_^25^ and 12R-Ba_4_CeMn_3_O_12_^30^), as well as in Ru and Ir analogs.^29^

Despite the interesting magnetism in these systems, there are few neutron diffraction studies that experimentally determine the magnetic structures of these 12R perovskites, possibly due to the difficulty in synthesizing these materials at high enough purities to enable magnetic experiments.^16^ As such, the complex interplay between local and extended structure and magnetism in these systems remains incompletely understood, and additional experimental and theoretical magnetostructural studies are needed.

We recently reported the synthesis and bulk AFM properties of two high-purity compounds Ba_4_*M*Mn_3_O_12_, (*M* = Ce, Pr) in the 12R polytype (Figure 1).^30^ As shown in Figure 1, 12R-Ba_4_CeMn_3_O_12_ (12R-BCM) is a simple AFM with a Néel temperature of *T*_N_ ≈ 7.75 K.^30^ However, the magnetic properties of 12R-Ba_4_PrMn_3_O_12_ (12R-BPM) are more complex: it exhibits a broad feature in the susceptibility data at approximately *T*_1_ ≈ 200 K, below which a small net moment appears. This is followed by a sharp peak at T_2_ ≈ 12.15 K likely indicative of long-range AFM order.^30^ The key difference between these two materials is the identity of the lanthanide that bridges between the Mn_3_O_12_ trimers: nonmagnetic Ce^4+^ vs magnetic 4f^1^ Pr^4+^.

Here, we further elucidate the effect of the interlayer cation on Mn^4+^ trimer coupling and on the magnetism in these systems by characterizing their magnetic structures using powder neutron diffraction (PND) measurements, X-ray absorption spectroscopy (XAS) measurements, and density functional theory (DFT)-based first-principles calculations. From the PND measurements, we determine the magnetic structure of these materials for the first time at various temperatures down to ca. 4 K. We demonstrate a collinear structure and spin canting in the Mn_3_O_12_ trimers induced by coupling to the paramagnetic Pr^4+^ interlayer ions. The calculated magnetic structures from first-principles calculations agree well with the experimentally derived magnetic structures, and the calculated density of states diagrams indicate both compounds are insulators. Our results can be generalized to the broader family of 12R systems, including those based on Ru_3_O_12_ and Ir_3_O_12_ trimers, advancing our understanding of the magnetostructural relationships in layered perovskite oxides and aiding future targeted functional materials discovery.

## Results and Discussion

### PND of 12R-BCM

**Figure 2:**
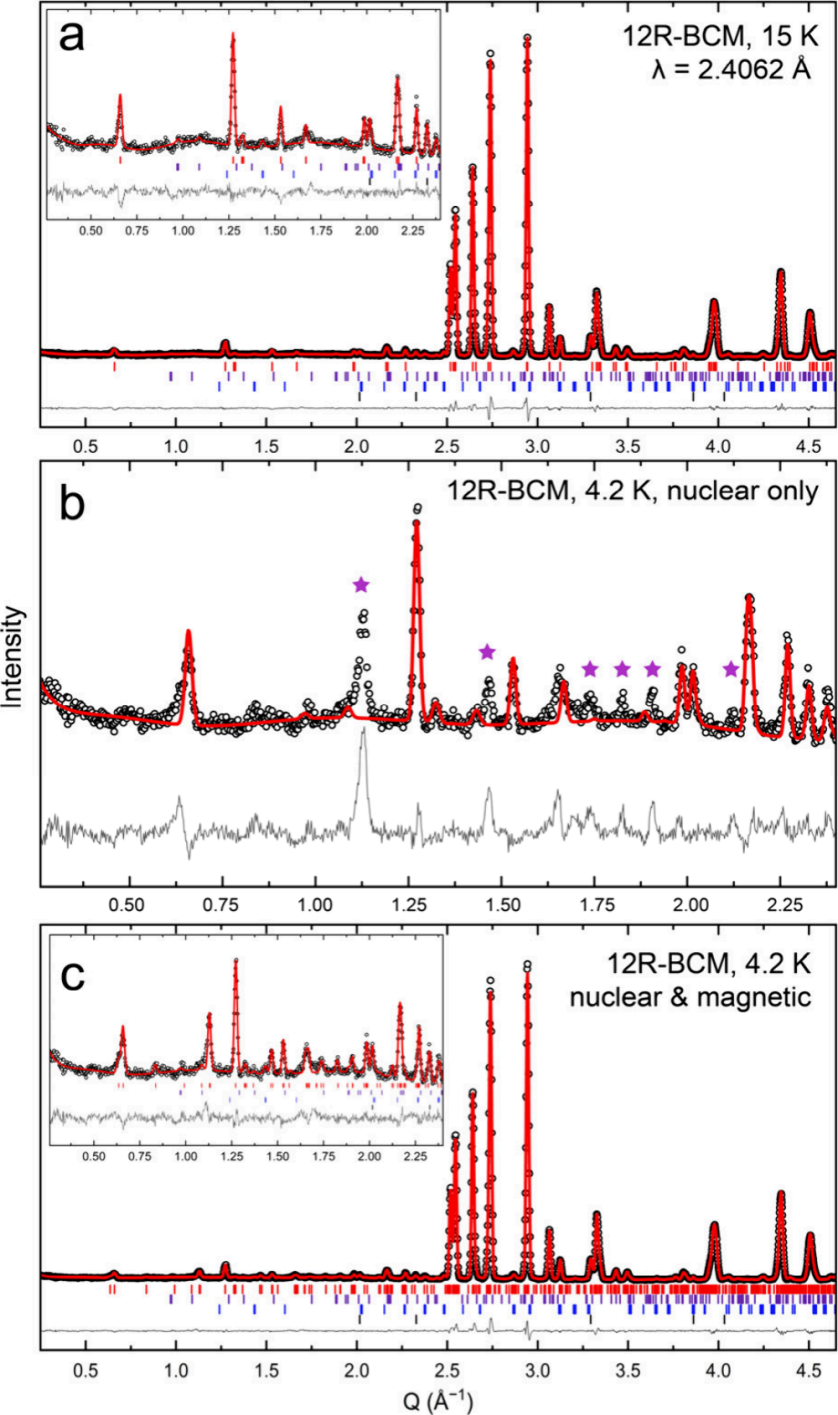
(a) The PND pattern collected at 15 K for 12R-BCM. The inset displays the low-*Q* region of the pattern. (b) The PND pattern collected for 12R-BCM at 4 K displays new Bragg peaks that are not well captured using a nuclear-only phase for the Rietveld refinement, as indicated by the purple stars. (c) The PND pattern at 4 K fitted using a nuclear and magnetic phase. For each, the vertical red, purple, blue, and black tick marks denote the *hkl* positions for 12R-BCM, Ba_4_Mn_3_O_10_, BaCeO_3_, and CeO_2_, respectively. Symbols are larger than or commensurate with their error bars which represent ±1σ. *R*_wp_ values for (a) and (c) are 4.267% and 3.921%, respectively. Data are also illustrated in Figures S1–S3.

To probe the low-temperature nuclear and magnetic structures, we collected powder neutron diffraction (PND) patterns at temperatures in the paramagnetic regime and in the magnetically ordered regimes for both compounds, as guided by the DC magnetic susceptibility data (maroon diamonds in Figure 1).^7,30^ The pattern collected at 15 K for 12R-BCM represents the material in the paramagnetic phase, and this pattern was analyzed first. We performed a Rietveld refinement against the PND pattern (Figure 2a, Figure S1). At 15 K, 12R-BCM crystallizes in the *R*3̅*m* space group, with *a* = 5.78299(5) Å, *c* = 28.5368(5) Å, and volume = 826.50(2) Å^3^. The resulting structural model is analogous to the published structure derived from refinement against SPXRD data at 100 and 300 K.^7,30^ The PND data revealed a phase purity of ≈93.3(1) % 12R-BCM with minor impurity phases of 3.0(1) wt % CeO_2_,^30^ 2.1(1) wt % BaCeO_3_,^31^ and 1.6(1) wt % Ba_4_Mn_3_O_10_^21^ consistent with the previous report.^30^ The crystal structures for the paramagnetic phases derived from PND and SPXRD for both 12R-BCM and 12R-BPM are deposited to the Cambridge Structural Database as deposition numbers 2378902–2378905.

We then applied this Rietveld model (including all nuclear phases) to the PND pattern collected for 12R-BCM at 4.2 K, below the Néel temperature, and inspected the resulting fit to the data to try to identify any possible new magnetic Bragg peaks in the diffraction pattern (Figure 2b). By eye, we identified at least six new Bragg peaks which were well-isolated from Bragg peaks from the nuclear phases, denoted by purple stars in Figure 2b. The new peaks occur at approximate scattering vector values of *Q* ≈ 1.13 Å^–1^, 1.46 Å^–1^, 1.74 Å^–1^, 1.82 Å^–1^, 1.91 Å^–1^, and 2.12 Å^–1^, respectively. We also identified additional features that may arise from magnetic scattering but that were not well isolated from the nuclear Bragg peaks, so we excluded these when trying to identify the propagation vector. We did not identify additional features in the 4.2 K data set (or upon inspection of the difference curve generated by subtracting the 15 and 4.2 K data sets) above *Q* ≈ 2.5 Å^–1^. Using the *k*-search functionality in FullProf^32^ based on this collection of well-separated magnetic Bragg peaks identified both above and in Figure 2b by purple stars, we find a propagation vector of  (equivalent to ). We then attempted to determine the magnetic structure of 12R-BCM using the magnetic space group formalism. Using ISODISTORT,^40,41^*k* point *Y* (, where ) and assuming magnetic moments on the Mn^4+^ ions only, we find two possible irreducible representations: Γ_1_ and Γ_2_, each having three potential solutions with maximal magnetic space groups 12.64, 15.91, and 5.17.

Next, we performed a combined nuclear and magnetic Rietveld refinement of the PND pattern collected for 12R-BCM at 4.2 K. We determined and initially fixed the lattice parameters of the nuclear phases using a Pawley fit to account for any thermal expansion. We then refined the background and the magnetic structure for each of the six possible solutions. After refining each of these solutions against the data, we find that the first irreducible representation (mY1, mk3t1), magnetic space group 15.91 gave the best fit to the magnetic Bragg peaks (Figure 2c). The resulting fit statistics of all magnetic refinements are given in Table 1. We omitted the two solutions obtained using magnetic space group 12.64 because they do not place magnetic moments on all Mn^4+^ cations by symmetry.

**Table 1:** Fit Statistic *R*_wp_ for Each Magnetic Structure Refinement against the Powder Data Collected at 4.2 K for 12R-BCM and 12R-BPM^a^

	IR	Mag. S.G.	*R*_wp_ (%)
12R-BCM	Γ_1_	15.91	3.921
		5.17	4.877
	Γ_2_	15.91	4.633
		5.17	4.699
12R-BPM	Γ_1_	15.91	6.073
		5.17	5.940
	Γ_2_	15.91	5.755
		5.17	5.909

a IR and Mag. S.G. stand for irreducible representation and magnetic space group, respectively.

**Figure 3:**
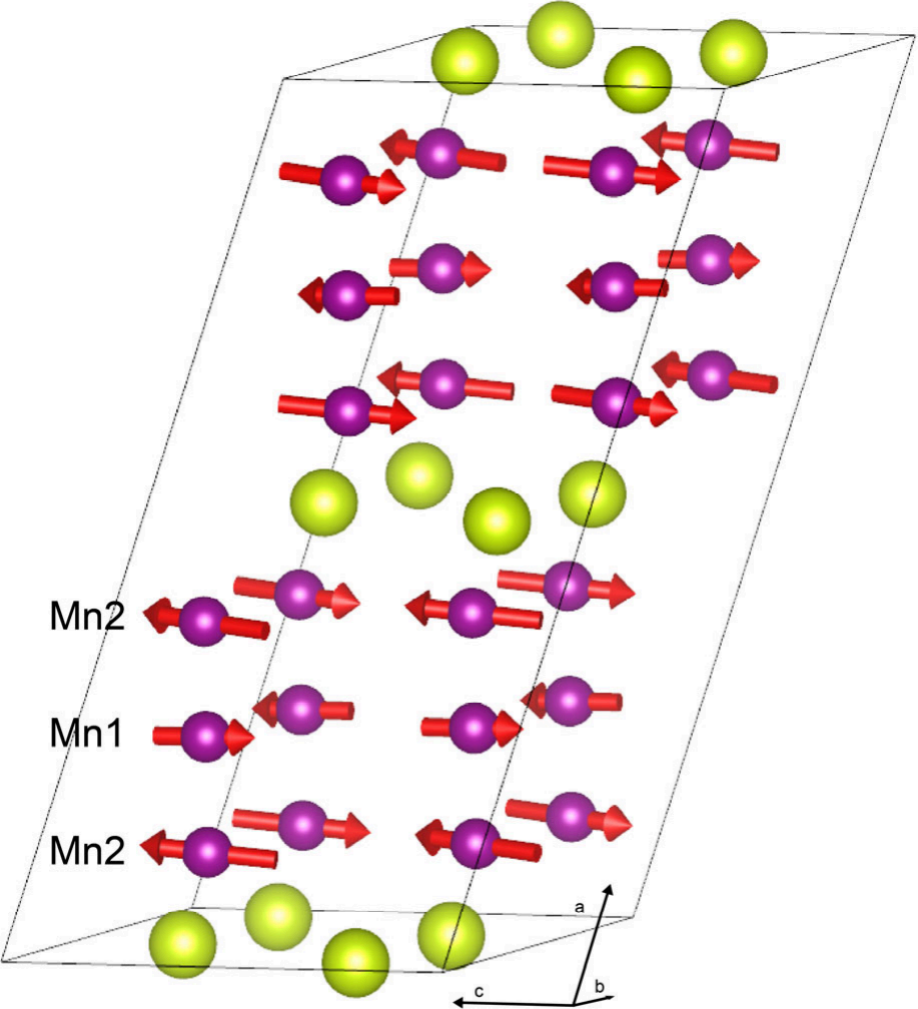
One magnetic unit cell of the experimentally derived magnetic structure of 12R-BCM as obtained from Rietveld refinements of the PND pattern collected at 4.2 K. Green and purple spheres depict Ce and Mn atoms, respectively, while red arrows denote magnitude and orientation of the spins.

We then systematically refined the background, the atomic coordinates for the 12R-BCM phase, and the lattice parameters for all phases. Occupancies were fixed according to the refinement of the 15 K data set. The final refinement fit to the data is illustrated in Figure 2c, and the resulting AFM structure is depicted in Figure 3. In the paramagnetic phase of 12R-BCM, layers in the crystallographic *ab*-plane contain octahedrally coordinated, face sharing, stacked Mn trimers. These are separated in the crystallographic *ab*-plane by nonmagnetic Ba cations, and in the *c*-axis direction by nonmagnetic Ce^4+^ and Ba cations. In the *R*3̅*m* structure, these trimers comprise two crystallographically distinct Mn^4+^ cations: the central Mn (Mn1) and a peripheral Mn (Mn2) that is duplicated about the central ion by symmetry. The monoclinic magnetic structure has the same two distinct Mn^4+^ ions in the asymmetric unit making up the trimers. Note that while the crystal structures do not change appreciably upon magnetic ordering, the definition of the unit cells do change because of the magnetic ordering, and the way that the unit cell is defined with respect to the Mn trimers changes going from the paramagnetic to the AFM phase (Figures 1 and 3; see crystal structures).

We find moments for the central and peripheral Mn^4+^ ions in the Mn_3_O_12_ trimers of 1.1(2) μ_B_ and 1.2(2) μ_B_, respectively. Note that ordered moments derived from PND measurements will be smaller than Curie–Weiss moments, in general, by ca. , so the difference between 1.2 μ_B_ (neutron) and 1.5 μ_B_ (Curie–Weiss fit between 47–100 K from ref (30)) is expected. The refined magnetic moments per Mn^4+^ ion give a net moment per formula unit =  =  = 2.0(4) μ_B_ per formula unit. This value is consistent with the value determined from previous Curie–Weiss fits of the magnetic susceptibility data.^7,30^ The peripheral moments are antiparallel to the moment from the central ion, and slightly canted about the moment from the central ion. By symmetry, the moment on the central ion is constrained to the *ac*-plane, whereas the moments on the peripheral Mn^4+^ ion cant slightly into the *bc*-plane, away from the central Mn^4+^ ion. This creates a magnetic structure in which the Mn^4+^ ions align antiparallel in the plane containing the Mn_3_O_12_ trimers, such that the net moment is zero but each Mn_3_O_12_ trimer has an unquenched moment supporting the hypothesis of cluster magnetism.^7,30^Table S1 specifies the magnetic structure of 12R-BCM at 4.2 K (see also crystal structures).

### PND of 12R-BPM

To investigate the effects of incorporating a magnetic ion at the M site, we then turned to the PND patterns collected for 12R-BPM. First, we analyzed the pattern collected at 250 K, in the paramagnetic phase of the material. After determining lattice parameters using a Pawley fit, we initialized the Rietveld refinement using the structural model determined from the SPXRD measurement (Figure S4). We achieved a good fit to the PND pattern (Figure S5) and a resulting structural model consistent with the literature.^32^ We find relative phase fractions from the Rietveld refinement of ≈89.5(1) wt % 12R-BPM, 5.9(1) wt % Ba_4_Mn_3_O_10_, and 4.6(1) wt % BaPrO_3_.

**Figure 4:**
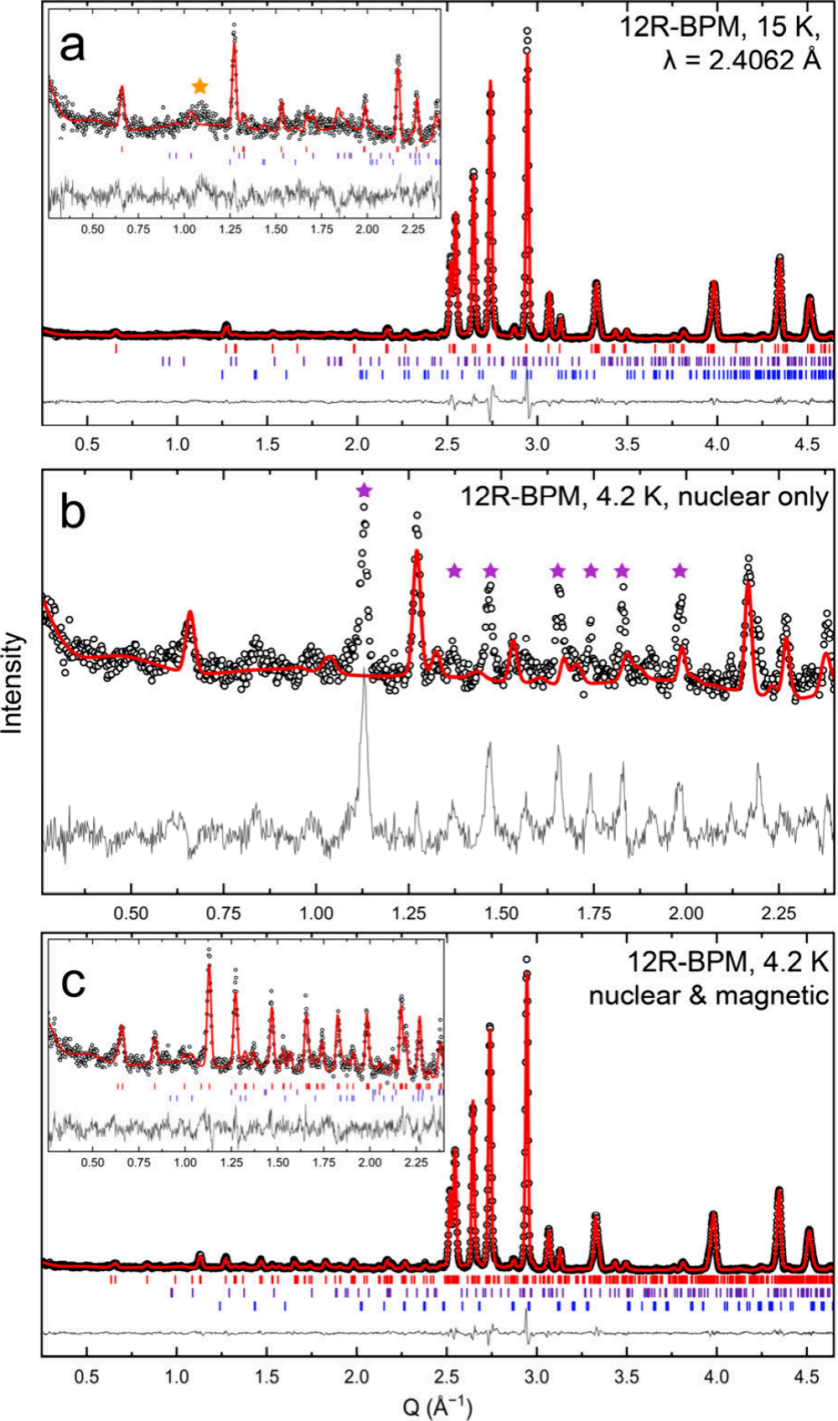
(a) The PND pattern collected at 15 K for 12R-BPM. The inset displays the low-*Q* region of the pattern. (b) The PND pattern collected for 12R-BPM at 4 K displays new Bragg peaks that are not well captured using a nuclear-only phase for the Rietveld refinement, as indicated by the purple stars. (c) The PND pattern at 4 K fitted using a nuclear and magnetic phase. For each, the vertical red, purple, and blue tick marks denote the *hkl* positions for the 12R-BPM, Ba_4_Mn_3_O_10_, and BaPrO_3_ phases, respectively. Symbols are larger than or commensurate with their error bars which represent ±1σ. *R*_wp_ values for (a) and (c) are 6.367% and 5.755%, respectively. Data are also illustrated in Figures S6–S9.

Given the plot of the DC susceptibility data for 12R-BPM as a function of temperature (Figure 1), we expected that we might resolve magnetic contributions in the PND patterns collected at 15, 9, and 4.2 K. To test this, we applied the structural model from the refinement against the 250 K data set to these data (Figure 4, Figures S6–S9). The data collected at 15 K closely resembles the data collected at 250 K. Inspection of the difference curve shows possible diffuse scattering at *Q* ≈ 1.1 Å^–1^, denoted by an orange star in Figure 4a in the inset and Figure S6. This may indicate local magnetic ordering of the Mn trimers at temperatures above the long-range magnetic ordering temperature, as hypothesized for 12R-Ba_4_NbMn_3_O_12_.^25^

In contrast, the PND patterns collected at 9 and 4.2 K display new, intense peaks which we tentatively assign as magnetic Bragg peaks (Figure 4 and Figure S6). By eye, the major difference between the data collected at 9 K and the pattern collected at 4.2 K is an increase in the intensity of this new set of Bragg peaks, further supporting this assignment. No additional peaks are observed between 9 and 4.2 K, and the relative intensities of the new peaks appear constant. The new set of Bragg peaks here is similar to the magnetic Bragg peaks identified for 12R-BCM at 4.2 K (Figure 2b), possibly hinting at similar magnetic structures. To determine the magnetic propagation vector for 12R-BPM, we selected new Bragg peaks in the pattern collected at 4.2 K that were well separated from nuclear Bragg peaks. These occur at scattering vector values of *Q* ≈ 1.13 Å^–1^, 1.46 Å^–1^, 1.65 Å^–1^, 1.74 Å^–1^, 1.81 Å^–1^, 2.12 Å^–1^, and 2.20 Å^–1^, respectively, as indicated by purple stars in Figure 4b. Running the k-search functionality in FullProf with this collection of Bragg peaks gives a most likely propagation vector of  (equivalent to ), the same propagation vector as identified for 12R-BCM. Using ISODISTORT, *k* point *Y* (, where ) and assuming magnetic moments on the Mn^4+^ and Pr^4+^ ions, we find the same two possible irreducible representations: Γ_1_ and Γ_2_, each having the same three potential solutions with maximal magnetic space groups 12.64, 15.91, and 5.17.

We then performed a Pawley fit of the PND pattern collected at 4.2 K for 12R-BPM to determine lattice parameters and peak shape parameters for the main and impurity phases. We fixed these in an initial Rietveld refinement along with the atomic coordinates and occupancies determined from the high temperature structure solution, and then iteratively tested the possible magnetic solutions against the data. Again, we find that, for both Γ_1_ and Γ_2_, the solutions from magnetic space group 12.64 fail to place moments on all the Mn^4+^ ions by symmetry, so these two solutions are excluded. The fit statistics for the other four potential solutions are listed in Table 1. Here, we find that Γ_2_, magnetic space group 15.91 yields the best fit to the data, although the fit statistics are more similar for 12R-BPM for the various fits compared to 12R-BCM, and differences to the fits to the magnetic Bragg peaks are somewhat minor (Figures S10 and S11). More detailed single crystal investigations may yield further insight into the magnetic structure. However, based on the *R*_wp_ values we posit that the Γ_2_ space group 15.91 fit best models the data. The atomic coordinates, displacement parameters and lattice parameters were then systematically refined against the data and a refinement consistent with the powder pattern was achieved (Figure S9).

**Figure 5:**
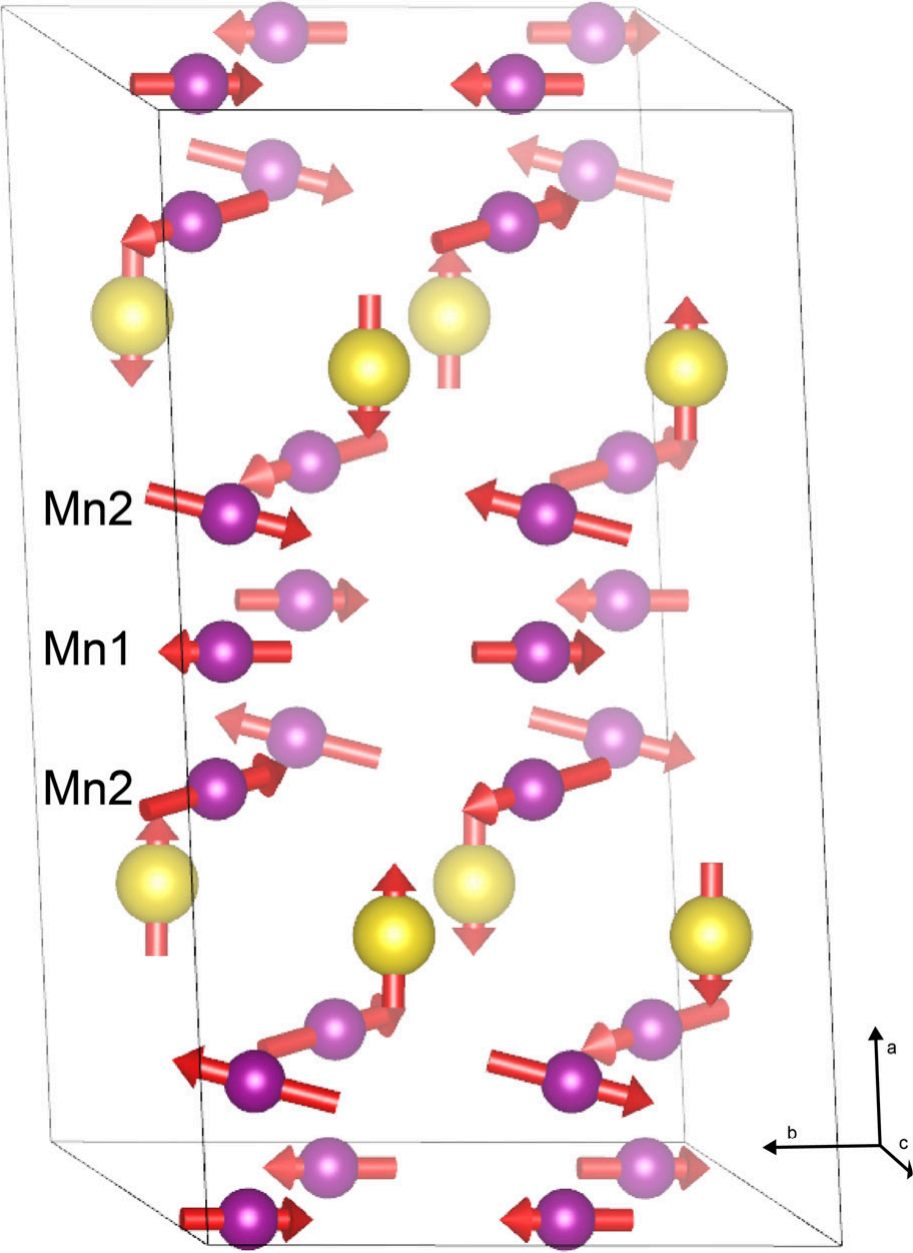
One magnetic unit cell of the experimentally derived magnetic structure of 12R-BPM as obtained from Rietveld refinements of the PND pattern collected at 4.2 K. Gold and purple spheres depict Pr and Mn atoms, respectively, while red arrows denote magnitude and orientation of the spins.

Finally, we performed combined nuclear and magnetic Rietveld refinements of the patterns collected at 15 and 9 K for 12R-BPM. We find for the pattern collected at 15 K that a model comprising a nuclear phase only for 12R-BPM yields a reasonable fit to the data (Figure 5a, Figure S7) without significant changes to the structure. Similarly, the pattern collected at 9 K was well modeled using a nuclear and magnetic phase for 12R-BPM derived from the refinement against the 4.2 K data (Figure S8). There are not significant changes in the nuclear structure. The orientation of the refined magnetic moments does not change appreciably but there is an increase in moment going from 9 to 4.2 K, as expected. Tables S2 and S3 specify the magnetic structures at 4.2 and 9 K, respectively.

### Comparison of Magnetic Structures

At 4.2 K, the crystal and magnetic structures locally at the Mn^4+^ trimers for 12R-BCM and 12R-BPM are qualitatively very similar. Both structures contain as the major magnetic feature isolated trimers of Mn^4+^ ions comprising three face-sharing MnO_6_ octahedra. A central octahedron, the Mn at the crystallographic (00^1^/_2_) position (Mn1; Wyckoff site 3*b*) is face-sharing with one MnO_6_ in the + direction and one MnO_6_ in the – direction, creating a central octahedron and two peripheral octahedra. The Mn in the peripheral octahedra occupies a crystallographic (00*z*) position (Mn2; Wyckoff site 6*c*) and as such there are two unique Mn sites in the asymmetric unit. Note that, while the magnetic structures for 12R-BCM and 12R-BPM are both described in magnetic space group 15.91, the different irreducible representations Γ_1_ and Γ_2_ generate different origins and arrangements of the asymmetric units within this space group. As such, the components of the moments in Tables S1–S3 are not directly comparable. For 12R-BCM, the Mn_3_O_12_ trimers are stacked parallel to the crystallographic *b*-axis based on the definition of the unit cell for the magnetic structure, whereas for 12R-BPM the Mn_3_O_12_ trimers are stacked along the crystallographic *a*-axis based on the definition of the unit cell for the magnetic structure (Figures 1, 3, and 5). Note that the orientation of the Mn trimers in the nuclear phase does not change relative to the other atoms in the structure; the only thing that changes is the definition of the unit cell.

Assuming pure Mn^4+^ and octahedral coordination, the Mn^4+^ ions are expected to display *S* = 3/2 ground states with a spin-only magnetic moment for each Mn^4+^ ion of ≈3.87 μ_B_. Yet in the PND data refinements we observed significantly suppressed ordered moments in 12R-BCM of 1.1(2) μ_B_ for Mn1 and 1.2(1) μ_B_ for Mn2, much smaller than expected and within error of each other. This is broadly consistent with the suppressed effective moment extracted from prior Curie–Weiss fits of magnetic susceptibility data collected on this sample (2.65(7) μ_B_ per formula unit or 1.53(4) μ_B_ per Mn in the 47–100 K fit range).^30^ A substantial part of this difference may be explained by the formation of antiferromagnetic Mn trimers with *net* effective spin 3/2, which would be expected to yield an effective moment of 2.23 μ_B_ per Mn (3.87 μ_B_ per formula unit), compared with 3.87 μ_B_ per Mn (6.71 μ_B_ per formula unit) for Mn^4+^ without trimerization. We also observe, however, a further reduction of both the ordered (neutron) and effective (Curie–Weiss) moment by a factor of ≈0.7 beyond the expected trimer values, suggesting that effects beyond trimer formation may be at play.

In 12R-BCM, the moment for Mn1 is restricted to the crystallographic *ac*-plane (perpendicular to the Mn trimer), whereas there is very slight canting out of plane for the Mn2 ions. By symmetry, the two peripheral Mn moments align antiparallel with the central Mn1 moment, such that each trimer has a net moment of 2.0(4) μ_B_, as described above. Each individual trimer can therefore be described as a magnetic cluster. The moments in adjacent trimers align antiparallel to create a collinear AFM structure, in agreement with the magnetic susceptibility data.^7,30^ This description agrees well with the hypothesized local magnetic structures for the trimers determined for Ba_4_NbMn_3_O_12_, Ba_4_Sn_1.1_Mn_2.9_O_12_, and Ba_4_SbMn_3_O_12_, despite the differences in Mn oxidation states.^9,11,16^

In contrast, in 12R-BPM, the Pr^4+^ ions are *J*_eff_ ≈ 1/2 and form an AFM lattice with spins aligned in an up–up–down–down pattern within the (101̅) plane, which alternates along the crystallographic *a*-axis direction. There is apparently coupling between the Mn trimers and the Pr^4+^ ions, and possibly stronger intratrimer coupling along the crystallographic *b*-axis (i.e., along the Mn trimers). This creates stronger canting out of plane, and at 4.2 K, the magnetic ions have moments of 1.72(7) μ_B_, 2.1(2) μ_B_, and 0.4(1) μ_B_, for Mn1, Mn2, and Pr, respectively. The net magnetization is  = 3.5(3) μ_B_ per formula unit. Notably, the canting follows an up–down–up–down pattern in the (203̅) plane. The moments on the peripheral Mn2 ions are slightly larger than the moments on the central Mn1 ions, possibly due to the added moment out of plane.

Interestingly, the local magnetism in the Mn trimers is quantitatively similar between 12R-BCM and 12R-BPM, yet 12R-BPM has a broad feature in the susceptibility data at ca. 200 K, much higher than the *T*_N_ for 12R-BPM (12.15 K). It is possible that there may be strong local magnetic correlations in both the 12R-BCM and 12R-BPM compounds between 100–300 K, although as discussed in our prior work, the feature at ca. 200 K in 12R-BPM could be consistent either with formation of strong short-range intratrimer correlations, with previous reports of non-Curie–Weiss behavior of Mn^4+^ ions above ≈200 K, or with a small amount of reduction of Mn^4+^ to Mn^3+^.^30^ We hypothesize that the inclusion of the paramagnetic Pr^4+^ ion perturbs the frustrated ground state and enables magnetic ordering at higher-*T* in 12R-BPM (*T*_N_ ≈ 12.15 K) compared to 12R-BCM (*T*_N_ ≈ 7.75 K). Further magnetic pair distribution function analysis may help shed light on the local magnetic correlations in these frustrated systems, although it is outside the scope of this investigation.

A review of existing literature on materials with the 12R structure reveals only one whose magnetic structure has been studied with PND: Ba_4_TbRu_3_O_12_, which is AFM with *T*_N_ = 24 K.^33^ Interestingly, this material displays a different magnetic structure of the M-site sublattice than 12R-BPM does: the Tb^4+^ moments are parallel with the Ru^4+^ moments, forming ferromagnetic planes of Tb^4+^ moments that are AFM to each other (i.e., A-type AFM). However, the Pr sublattice in 12R-BPM exhibits a C-type AFM structure. In addition, no canting is observed in Ba_4_TbRu_3_O_12_, so we hypothesize canting likely occurs in 12R-BPM because the Pr^4+^ moments are orthogonal to the Mn^4+^ moments.

### Electronic Structure and Calculations

Next, we characterized the electronic structures in 12R-BCM and 12R-BPM experimentally and computationally. Based on the crystal structures and previous work on similar samples,^34,35^ we expect Mn^4+^ and Ce^4+^ in our 12R-BCM sample—and by extension Mn^4+^ and Pr^4+^ in 12R-BPM, which has not yet been studied with XAS. However, we observe that the behavior at *T*_1_ ≈ 200 K in the magnetic susceptibility of 12R-BPM is like that of a few other 12R materials (Ba_4_NbMn_3_O_12_ and Ba_4_Fe_3_NiO_12_) and may be related to minor reduction of Mn^4+^ to Mn^3+^.^25,28^ The metal oxidation states are especially important to quantify because past work on compounds with oxidation states other than +4 at the M site (i.e., as in 12R-Ba_4_NbMn_3_O_12_ and 12R-Ba_4_TaMn_3_O_12_^26^) shows that the M oxidation states can have a large impact on the number of unpaired electrons in the Mn trimers and thus on the bulk magnetism. Unfortunately, these differences in electronic structure at the M sites hinder direct comparisons across this family of compounds. To verify that comparisons between 12R-BCM and 12R-BPM here are valid, and to probe the oxidation states of both samples more directly, we conducted soft X-ray absorption spectroscopy (XAS) at the Mn *L*_3_-edge and the O *K*-edge on both 12R-BCM and 12R-BPM, along with hard XAS measurements at the Mn *K*-edge and Pr *L*_3_-edge on 12R-BPM (Figures S13–S16). The Mn *L*-edge spectra are very similar for both materials and are in good agreement with a predominantly Mn^4+^ state based on comparison with prior literature on both 12R-BCM and a MnO_2_ standard (Figure S13).^34,36^ A subtle difference between the two spectra is present as the absorption onset feature for 12R-BPM is slightly shifted to lower energy in the *L*_3_ peak, which may indicate a very small presence of reduced Mn in 12R-BPM relative to 12R-BCM. The Mn *K*-edge spectrum for 12R-BCM (Figure S14) shows that it is more highly oxidized than Mn^3+^ standards, consistent with a primarily Mn^4+^ state.

**Figure 6:**
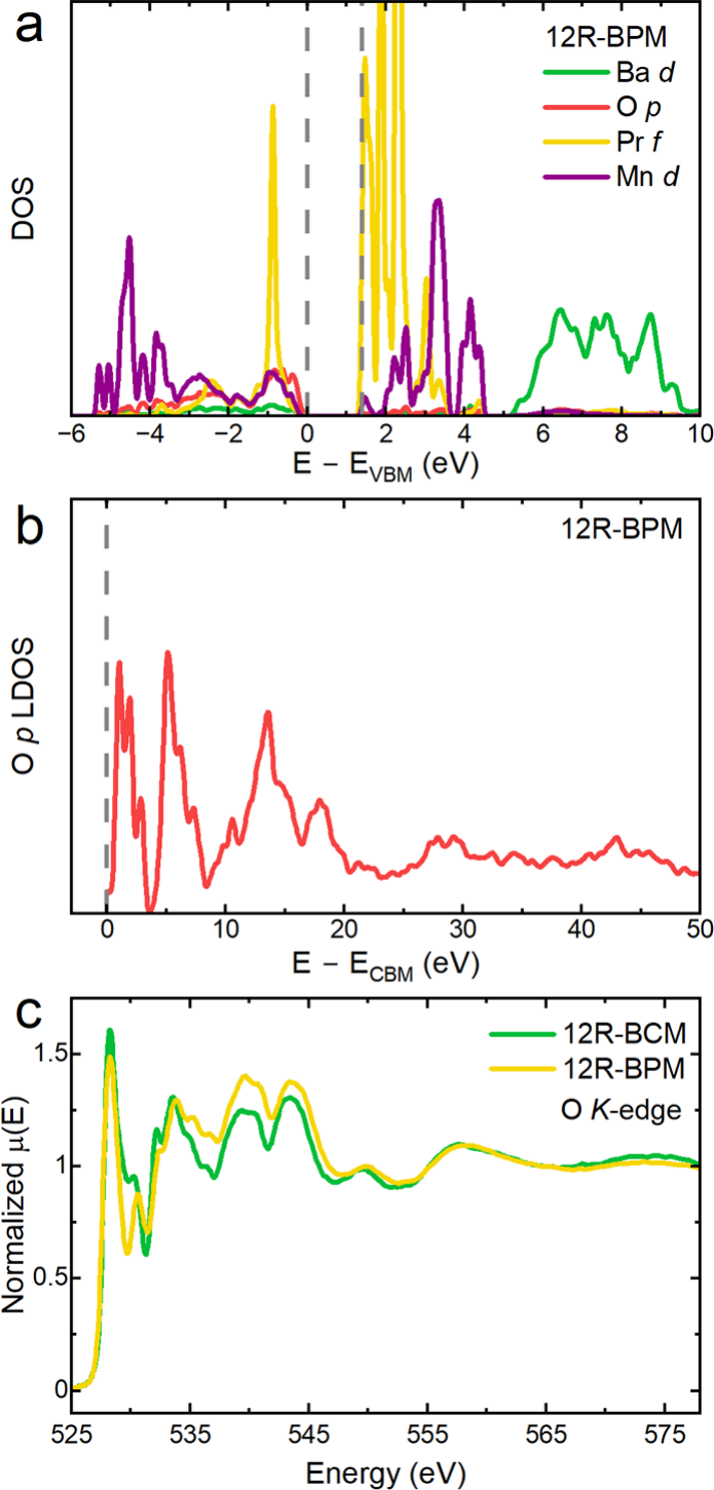
DFT- and XAS-based investigations of the electronic structure for 12R-BPM. (a) DFT+*U* calculated partial DOS of 12R-BPM in the vicinity of the band gap reveals insulating behavior with a band gap of 1.40 eV. (b) O-p LDOS of 12R-BPM up to 50 eV above the CBM. (c) O *K*-edge XAS spectra of 12R-BCM and 12R-BPM.

More significant differences are present in the comparison of the more complex O *K*-edge data (Figure 6c, Figure S15). Within the O *K*-edge spectra, the peaks present between 525 and 535 eV are pre-edge features characteristic of an O 1s excitation to hybridized O 2p and Mn 3d states.^34,35,37^ Within this regime, 12R-BPM has lower intensity peaks at ≈528 and 531–532 eV relative to 12R-BCM. We interpret the signal between ca. 535 to 547 eV as originating from O 1s transitions to O 2p – Ln 5d and O 2p – Mn 4sp states, where 12R-BPM has higher intensity relative to 12R-BCM. A literature study of several LnTMO_3_ structures where Ln = lanthanide ions and TM = transition ions found that the ratio of signal below ≈533 eV (O 1s to O 2p – TM 3d transition) to above ≈533 eV (O 1s to O 2p – Ln 5d and O 2p – TM 4sp transitions) correlated to both the number of TM 3d electrons and the net oxidation state of the TM due to both factors’ impact on the degree of covalent hybridization between the O 2p and TM 3d.^35^ Comparing 12R-BCM to 12R-BPM, the number of TM 3d electrons does not vary, making the difference in pre- and postedge signal most likely correlated to a slight difference in net oxidation state of the Mn. 12R-BPM has a slightly lower apparent ratio of pre- to postedge signal, indicative of a slightly lower net oxidation state of Mn than that in 12R-BCM.

Hard XAS data for 12R-BPM at the Pr *L*_3_-edge (Figure S16) indicate that some minor amount of Pr^3+^ may be present—although accurate quantification is difficult—and that the Pr–O bonding is more covalent than ionic. It is possible that the different electronic properties of Pr relative to Ce are responsible for the subtle differences in the absorption spectra and subsequent assignment to net oxidation state; however, possible differences in sample synthesis and processing history that could yield different oxygen vacancy concentrations cannot be discounted. Therefore, the origin of the difference in Mn oxidation state between the materials cannot be definitively assigned here.

Lastly, we performed first-principles DFT+U calculation for the collinear magnetic structures of 12R-BCM and 12R-BPM. Within the constraints of the small 40-atom simulation cell, the magnetic structures agree with the experimental characterization in that they exhibit Mn-trimers with alternating spins as well as in-plane AFM ordering of the trimers and of the Pr spins in the case of 12R-BPM. Crystal structures derived from DFT calculations are available. The calculated band gaps are 1.96 eV for 12R-BCM and 1.40 eV for 12R-BPM, but these DFT+U gaps are probably significantly underestimated. The local magnetizations of 2.9, 1.2, and 0.0 μ_B_ on Mn, Pr, and Ce, respectively, in the collinear calculation are consistent with the oxidation states Mn^4+^ (high-spin 3d^3^), Pr^4+^ (4f^1^), and Ce^4+^ (4f^0^). Supplemental noncollinear calculations show that the magnetizations are not significantly modified by spin–orbit coupling. The only appreciable orbital moment is observed at the Pr site (0.5 μ_B_).

To test the hypothesis of cluster magnetism, we further calculated the (collinear) spin-flip energies inside the Mn trimers. The magnetization of the alternating spin configuration is 3.0 μ_B_/trimer. A flip of the central Mn results in parallel alignment of all three spins (8.8 μ_B_/trimer), whereas a flip of a peripheral Mn maintains the lower magnetization (2.9 μ_B_/trimer). The energy costs of the flips are about 60 and 40 meV, respectively, in both BCM and BPM. These values suggest that the alternating-spin trimers mostly act as a magnetic unit below room temperature, supporting the hypothesis that the susceptibility behavior is governed by cluster magnetism of high-spin Mn^4+^.

Figure 6a shows the calculated partial density of states (DOS) in the vicinity of the Fermi level. The occupied Pr-4f state lies about 1 eV below the valence band maximum (VBM), whereas the unoccupied 4f states form the conduction band minimum (CBM) of 12R-BPM. The unoccupied Mn-3d states begin about 0.5 eV above the CBM, and the empty Pr- and Ba-5d orbitals extend over a range of about 3–10 and 4–9 eV with respect to the CBM. Figure 6b shows the O-p partial DOS up to 50 eV above the CBM, which approximates the XAS for the O *K*-edge by accounting for the selection rule Δ*l* = 1 for dipole transitions for the excitation from the O-1s state. Even without explicit modeling of optical matrix elements and core-hole excitation, this partial DOS often gives a decent representation of the XAS.^38^ Due to hybridization effects, the O-p partial DOS clearly reflects the above-mentioned Pr, Mn, and Ba orbitals up to about 10 eV above the CBM. Above that, the DOS structure reflects interactions with the full spectrum of higher-lying empty atomic orbital shells. Overall, the DOS structure shows a clear correspondence to the features observed in the experimental O *K*-edge spectra (Figure 6c), validating the theoretical description of 12R-BPM.

## Conclusions

In conclusion, we determined the magnetic structures for hexagonal perovskites 12R-BCM and 12R-BPM using PND, adding to the very short list of known magnetic structures in this otherwise relatively well-studied family of quantum materials. Both 12R-BCM and 12R-BPM order antiferromagnetically with a propagation vector of , and both materials can be described in magnetic space group 15.91. Interestingly, this propagation vector is different from the predicted vector found for 12R-Ba_4_NbMn_3_O_12_ and 12R-Ba_4_TaMn_3_O_12_.^26^ While 12R-BCM and 12R-BPM are described by the same magnetic space group, different irreps provide the best fit for each, causing their magnetic structures to be slightly different, especially at the interlayer Ce/Pr site. In 12R-BPM, the Pr^4+^ ions order in a C-type AFM lattice in an up–up–down–down pattern that alternates between layers. The magnetic structures for 12R-BCM and 12R-BPM differ in the degree of canting of the spins on the peripheral Mn^4+^ ions, with canting observed for 12R-BPM caused by coupling to the interlayer Pr^4+^ ion. This detailed understanding of the crystal structures of 12R-BCM and 12R-BPM should be considered to help inform possible magnetic structures predictions for other 12R compounds, such as 12R-Ba_4_NbMn_3_O_12_, where three somewhat different structures have been suggested in the literature.^25−27^ Moreover, the experimentally derived magnetic structures for 12R-BCM and 12R-BPM highlight how interlayer (dia)magnetic ions can affect coupling and canting in otherwise low dimensional systems, which is important when considering materials design for targeted magnetic or spintronic functionalities.

## Experimental Section

### Materials

We synthesized large, high-purity polycrystalline samples of 12R-Ba_4_CeMn_3_O_12_ (“12R-BCM”) and 12R-Ba_4_PrMn_3_O_12_ (“12R-BPM”) following solid state and sol gel synthesis methods, respectively, as detailed in previous work.^30^ We employed two different synthetic methods as we found these yielded the highest purity bulk samples for each of the compounds, respectively. These samples crystallize in the *R*3̅*m* space group down to at least 100 K. We previously assessed the bulk purity of the 12R-BCM sample using synchrotron powder X-ray diffraction (SPXRD), finding a phase purity of 98.4(1) wt %.^30^ Here, we used Rietveld refinements of SPXRD data of this 12R-BPM sample collected at 100 K at beamline 28-ID-2 at NSLS-II, BNL to determine the nuclear structure, with *a* = 5.79184(4) Å, *c* = 28.5362(4) Å, and volume = 829.01(1) Å^3^. Minor impurity phases of Ba_4_Mn_3_O_10_, BaPrO_3_ were observed, and the phase purity of the 12R polytype was 95.8(1) wt %. To our knowledge, these are the highest-purity samples ever reported, and are suitable for magnetic investigations.^30^ In this work, we measured the nuclear structures of both materials down to 4 K with powder neutron diffraction (PND) measurements, observing little change in the nuclear structures. Rietveld refinements of the PND data show that the *R*3̅*m* symmetry is maintained down to the lowest temperature measured and that both samples consist primarily of the 12R polytype (Figures 2 and 4; Figures S1 and S7; Tables S8, S9, S16, and S17), consistent with the SPXRD results.

### Synchrotron Powder X-ray Diffraction

≈50 mg of black, polycrystalline powder of 12R-BPM was loaded into a quartz capillary (inner diameter 1 mm) in air and mounted on the goniometer at the high-resolution powder diffractometer 28-ID-2 at NSLS-II, BNL (λ = 0.1821 Å). Temperature control was achieved using a stream of heated, flowing N_2_ gas over the capillary. A synchrotron powder X-ray diffraction (SPXRD) pattern was collected for 60 s at *T* = 100 K while the capillary was spinning. Scattered photon intensity was measured using a PerkinElmer XRD 1621 Digital Imaging Detector and data reduction was carried out using Dioptas.^39^ Pawley fits and subsequent Rietveld refinements were conducted using the TOPAS Academic software package.^40−42^

### Powder Neutron Diffraction

4.2 g of black polycrystalline powder 12R-BCM and 2.8 g of black polycrystalline powder 12R-BPM were loaded into separate vanadium cans (inner diameter 6 mm) with packed quartz wool and aluminum lid with a bore for exchange gas. The samples were loaded into a closed-cycle refrigerator with autosampler then mounted on the sample stage at the high-resolution powder diffractometer HB-2A, at the High Flux Isotope Reactor (HFIR), Oak Ridge National Laboratory (ORNL). The samples and sample environment were evacuated under dynamic vacuum for ca. 90 min using a turbomolecular vacuum pump (base pressure of ca. 5 × 10^–6^ mbar achieved) and then the sample space was partially pressurized with ≈200 mbar He heat exchange gas. The samples were cooled to 4.2 K and data were collected using an incident neutron beam with λ = 2.4062 Å, generated using a Ge(113) monochromator and open–open-12′ collimation. Powder neutron diffraction (PND) patterns were collected for 4 h for each pattern for 12R-BPM and for 6 h each for 12R-BCM and at temperatures of 4.2 and 15 K for both samples. Additional patterns were collected at 9 and 250 K for 12R-BPM. The zero error and wavelength for the instrument were calibrated by measuring a LaB_6_ + Si standard reference. The data were analyzed in TOPAS Academicas well as ISODISTORT.^42,43,^

### Soft X-ray Absorption Spectroscopy

Soft XAS data acquisition was performed at the Stanford Synchrotron Radiation Lightsource (SSRL) at beamline 10–1. Samples of 12R-BCM and 12R-BPM were mounted on carbon tape by brushing a thin layer of powder onto the tape surface. For fluorescence yield measurements (O *K*-edge) a Silicon Diode AXUV100 detector was used; for total electron yield measurements (Mn *L*-edge) a Channeltron detector was used. A reference spectrum was collected on an in-house standard comprised of an amalgamation of commonly measured metal oxides. All three data types were collected simultaneously for each edge. Three spots were measured on each sample, with two consecutive measurements collected for each spot. The resulting six spectra were energy-scale aligned and averaged in Athena.^44^ Background correction and normalization was performed in Igor Pro 9.01 (Wavemetrics). In the case of the Mn *L*-edge, normalization was performed at the *L*_3_ peak maximum. For the O *K*-edge, normalization was performed by estimating the postedge intensity from an average of the values across the range of 565–575 eV.

### Hard X-ray Absorption Spectroscopy

Hard XAS at the Mn *K*-edge and the Pr *L*_3_-edge was performed on 12R-BPM at beamline 6-BM at the National Synchrotron Light Source (NSLS-II) at Brookhaven National Laboratory (BNL). The beamline was equipped with a paraboloid collimating mirror coated with rhodium, a monochromator utilizing a Si(111) crystal, and a flat mirror designed for rejecting harmonic frequencies. Spectroscopic references, including MnO powder (Sigma-Aldrich) and Mn_2_O_3_ (99.9%, Sigma-Aldrich), were first measured in fluorescence mode before each scan on the sample for the energy calibration. The sample was diluted with BN. Spectra were collected at room temperature in transmission mode. Three scans at the Mn *K*-edge and seven scans at the Pr *L*_3_-edge were collected and averaged to further improve the signal-to-noise ratio of the absorption spectra. Data were processed in Athena.^44^

## Computational Section

### First-Principles Density Functional Theory (DFT) Calculations

DFT total energy calculations were performed using the projector augmented wave (PAW) method^45,46^ as implemented in the VASP (Vienna Ab-initio Simulation Package) code.^47^ The generalized gradient approximation (GGA) of Perdew et al.^48^ was used for DFT exchange and correlation along with a Hubbard U correction term^49^ with parameter U–J of 3 eV for Mn-d and 1.5 eV for Ce, Pr-d and 2 eV for Ce, Pr-f orbitals. The PAW potentials “Ba_sv,” “O_s,” “Mn,” “Ce_h,” and “Pr” distributed with VASP were used with a plane wave energy cutoff of 380 eV, consistent with the computational approach employed in previous DFT studies on transition metal oxides.^50−52^ We performed DFT+U calculations on a 12R phase (space group *R*3̅*m* #166) of BCM and BPM, using a gamma-centered 2 × 6 × 2 k-mesh for density of states (DOS) calculations. The local magnetizations and orbital-resolved partial DOS was determined with integration radii of 0.66, 1.50, 2.15, 2.03, and 2.04 Å for O, Mn, Ba, Pr, and Ce, respectively, taken as the covalent radii of ref.^53^ A Gaussian smoothing with σ = 0.05 and 0.20 eV was applied to the DOS in Figure 6a,b, respectively. The calculations were based on the previously determined lowest-energy collinear AFM configuration within a 40-atom cell for BCM.^54^ For BPM, we additionally find preferential AFM ordering of the Pr atoms. All figures illustrating crystal structures made use of the software package VESTA.^55^

## Supporting Information Available

Additional plots of the diffraction patterns and fits, XAS data, and tables containing crystallographic information (PDF)
