# Supplementary material for: Interlayer Ions Control Spin Canting in Low-Dimensional Manganese Trimers in 12R-Ba4MMn3O12 (M = Ce, Pr) Layered Perovskites
Source: Inorg Chem. 2024 Dec 7;63(51):24176–86. doi: 10.1021/acs.inorgchem.4c03915 (PMC11709229; doi:10.1021/acs.inorgchem.4c03915)
Supplement: Supplementary file 1 — ic4c03915_si_001.pdf [file ic4c03915_si_001.pdf]

Supporting Information for:

Interlayer ions control spin canting in low-dimensional manganese trimers in 12R-$Ba_4MMn_3O_{12}$ (M = Ce, Pr) layered perovskites

Corlyn E. Regier,[a] Shaun O'Donnell,[b] Anuj Goyal,[b,c] Michael J. Dzara,[b] James Eujin Park,[d] Robert T. Bell,[b] Morgan J. Kramer,[f,g] Joseph A. M. Paddison,[e] Sarah Shulda,[b] David S. Ginley,[b] Danielle R. Yahne,[e] Stephan Lany,[b] Rebecca W. Smaha,[b*] Ryan A. Klein[b,f*]

[a] *Department of Chemistry, Colorado State University, Fort Collins, Colorado 80523, USA*
[b] *Materials, Chemical, and Computational Sciences Directorate, National Renewable Energy Laboratory, Golden Colorado 80401, USA*
[c] *Department of Materials Science and Metallurgical Engineering, IIT Hyderabad, Kandi, Sangareddy, Telangana 502284, India*
[d] *Sandia National Laboratories, PO Box 5800, Albuquerque, New Mexico 87185, USA*
[e] *Neutron Scattering Division, Oak Ridge National Laboratory, Oak Ridge, Tennessee 37831, USA*
[f] *Center for Neutron Research, National Institute for Standards and Technology, Gaithersburg, Maryland 20899, USA*
[g] *Department of Chemical and Biomolecular Engi-neering, University of Delaware, Newark, DE, 19716, USA*
[*] *corresponding authors:* ryan.klein@nrel.gov rebecca.smaha@nrel.gov

Inorganic Chemistry

Table of Contents

**Additional details of refinement of SPXRD pattern**		**S3**
**Additional XAS details**		**S3**
**Figure S1**	Rietveld refinement of the PND pattern obtained for 12R-BCM at 15 K	**S4**
**Figure S2**	Magnetic Bragg peaks in the pattern collected for 12R-BCM at 4.2 K	**S5**
**Figure S3**	Rietveld refinement of the PND pattern obtained for 12R-BCM at 4.2 K	**S6**
**Figure S4**	Rietveld refinement of the SPXRD pattern obtained for 12R-BPM at 100 K	**S7**
**Figure S5**	Rietveld refinement of the PND pattern obtained for 12R-BPM at 250 K	**S8**
**Figure S6**	PND patterns collected for 12R-BPM at 250 K, 15 K, 9 K, and 4.2 K	**S9**
**Figure S7**	Rietveld refinement of the PND pattern obtained for 12R-BPM at 15 K	**S10**
**Figure S8**	Rietveld refinement of the PND pattern obtained for 12R-BPM at 9 K	**S11**
**Figure S9**	Rietveld refinement of the PND pattern obtained for 12R-BPM at 4.2 K	**S12**
**Figure S10**	Low Q region of Rietveld refinement for 12R-BPM at 4.2 K using Γ1 SG 5.17	**S13**
**Figure S11**	Low Q region of Rietveld refinement for 12R-BPM at 4.2 K using Γ2 SG 5.17	**S14**
**Figure S12**	Magnetic and nuclear crystal structures for 12R-BCM and 12R-BPM	**S15**
**Figure S13**	XAS spectra of Mn *L*-edge for 12R-BCM and 12R-BPM	**S16**
**Figure S14**	XAS spectrum of Mn *K*-edge for 12R-BPM	**S17**
**Figure S15**	XAS spectra of O *K*-edge for 12R-BCM and 12R-BPM	**S18**
**Figure S16**	XAS spectrum of Pr *L*-edge for 12R-BPM	**S19**
**Table S1**	Refined magnetic moments for 12R-BCM at 4.2 K	**S20**
**Table S2**	Refined magnetic moments for 12R-BPM at 4.2 K	**S20**
**Table S3**	Refined magnetic moments for 12R-BPM at 9 K	**S20**
**Table S4**	Crystallographic information from Rietveld refinement of PND data, 12R-BCM, 15 K	**S21**
**Table S5**	Crystallographic information from Rietveld refinement of PND data, 12R-BCM, 15 K	**S21**
**Table S6**	Crystallographic information from Rietveld refinement of PND data, 12R-BCM, 4.2 K	**S22**
**Table S7**	Crystallographic information from Rietveld refinement of PND data, 12R-BCM, 4.2 K	**S22**
**Table S8**	Crystallographic information from Rietveld refinement of SPXRD data, 12R-BPM, 100 K	**S23**
**Table S9**	Crystallographic information from Rietveld refinement of SPXRD data, 12R-BPM, 100 K	**S23**
**Table S10**	Crystallographic information from Rietveld refinement of PND data, 12R-BPM, 250 K	**S24**
**Table S11**	Crystallographic information from Rietveld refinement of PND data, 12R-BPM, 250 K	**S24**
**Table S12**	Crystallographic information from Rietveld refinement of PND data, 12R-BPM, 15 K	**S25**
**Table S13**	Crystallographic information from Rietveld refinement of PND data, 12R-BPM, 15 K	**S25**
**Table S14**	Crystallographic information from Rietveld refinement of PND data, 12R-BPM, 9 K	**S26**
**Table S15**	Crystallographic information from Rietveld refinement of PND data, 12R-BPM, 9 K	**S26**
**Table S16**	Crystallographic information from Rietveld refinement of PND data, 12R-BPM, 4.2 K	**S27**
**Table S17**	Crystallographic information from Rietveld refinement of PND data, 12R-BPM, 4.2 K	**S27**
**References**		**S28**

Analysis of synchrotron powder X-ray diffraction pattern—12R-BPM:

Initial Pawley fits indicated that this material crystallizes in the $R\overline{3}m$ space group, consistent with previous reports, with a = 5.79184(4) Å, c = 28.5362(4) Å, and volume = 829.01(1) Å^3. We used the published structure for 12R-BPM to initialize the Rietveld refinement and observed small, unindexed peaks remaining in the pattern, which arise from minor $Ba_4Mn_3O_{10}$, $BaPrO_3$, and PrO_2 impurity phases. We then refined atomic occupancies for 12R-BPM and found that the occupancies refined to one within error. We fixed these at unity for all subsequent refinements. We systematically refined atomic coordinates, lattice parameters, background terms, and displacement parameters until we arrived at a refinement which is consistent with the SPXRD data (Fig. 4). The resulting structure is directly analogous to the structure for 12R-BPM presented in the literature. No restraints or constraints were used during the refinement. Given the negligible intensity of the scattering from the minority phases, the atomic coordinates for the impurity phases were not refined.

Additional XAS Details

The Mn K-edge spectrum of 12R-BPM compared to several measured Mn standards (Fig. S12) indicates Mn is predominantly in the 4+ state. Additionally, there are several smaller peaks occurring before the main edge at ~6537 eV and ~6544 eV that qualitatively match with the Mn^{3+}standards. Therefore, this data suggests that Mn is predominantly Mn^{4+} with minor amounts of Mn^{3+} in 12R-BPM.

Figure S14a shows the hard XAS spectra on 12R-BPM at the Pr L_3-edge. The second derivative of the XAS spectrum (Fig. S14b) reveals four clear peaks which have been labeled A1, A2, B, and C in accordance with prior work from Hu et al.[1] Peaks A1 and A2 were assigned as Pr^{4+} core-excited final states $2p4f^15d^*$, where 2p denotes a hole in the 2p orbital and 5d* denotes the excited electron in the 5d orbital. Peak B was assigned to a Pr^{4+} $2p4f^25d^*L$ state, where L denotes a hole in the anion ligand orbital. The relative weights of peaks A to peak B are used to infer the relative ionicity of Pr^{4+} in the compound, where the larger weight of peak B relative to the sum of peaks A indicates a more covalent nature. Peak C is assigned to Pr^{3+}. Peak C is relatively large and indicates that there is a significant amount of Pr^{3+} in the compound. The multitude of peaks in the pre-edge region have not been fully assigned. Hu et al. labeled a peak at ~5961 eV as a dipole-forbidden $2p3/2 \rightarrow 4f$ transition,[1] but the other peaks in our data do not appear in their spectra. The overall conclusion drawn from these data is that the Pr in 12R-BPM is a mixture of Pr^{4+} and Pr^{3+} and the bonding is more covalent than ionic.

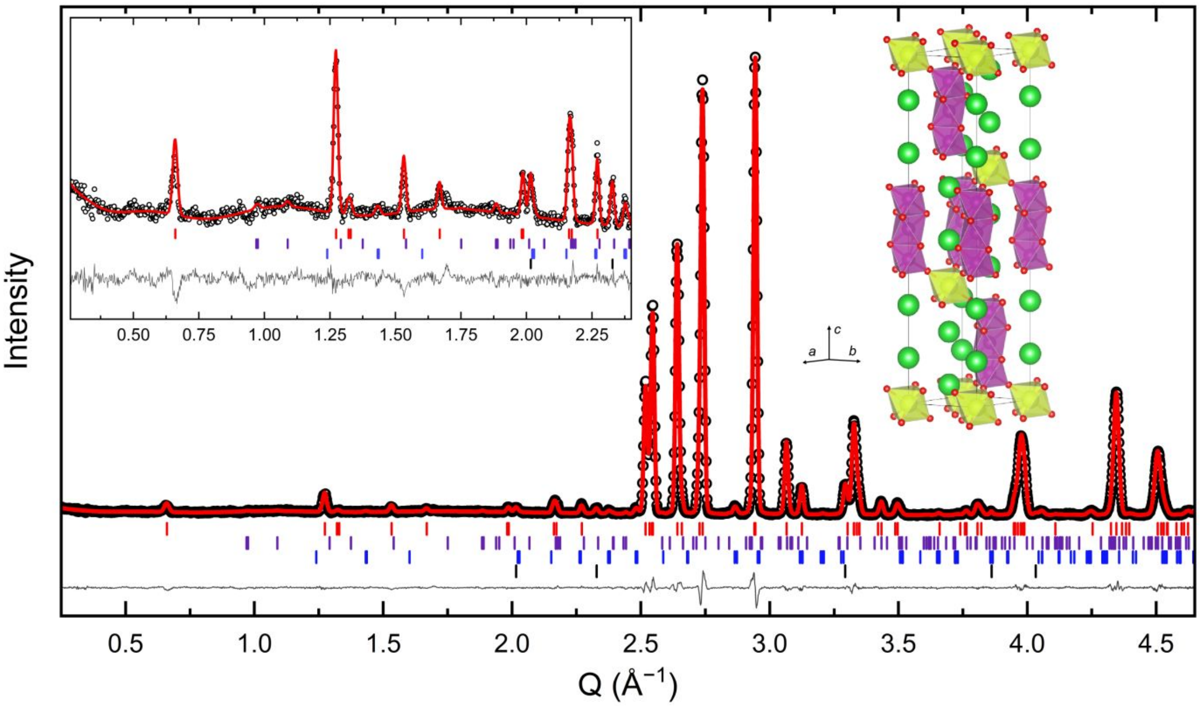

Figure S1 | Rietveld refinement of the PND pattern obtained for 12R-BCM. T = 15 K, λ = 2.4062 Å, R_{wp} = 4.267 %, R_p = 3.361 %, R_{exp} = 2.365 %, GOF = 1.804. The black circles, red curve, and grey curve denote the raw data, the Rietveld refinement curve, and the difference curve, respectively. The vertical red, purple, blue, and black tick marks denote the *hkl* positions for 12R-BCM, $Ba_4Mn_3O_{12}$, $BaCeO_3$, and CeO_2, respectively. Symbols are larger than or commensurate with their error bars which represent $\pm 1\sigma$. The inset (left) illustrates the data and fit in the low-Q region of the pattern. The inset (right) displays one unit cell of the resulting crystal structure. Green and yellow polyhedra represent Ce and Mn coordination environments, while red and green spheres depict O and Ba atoms, respectively.

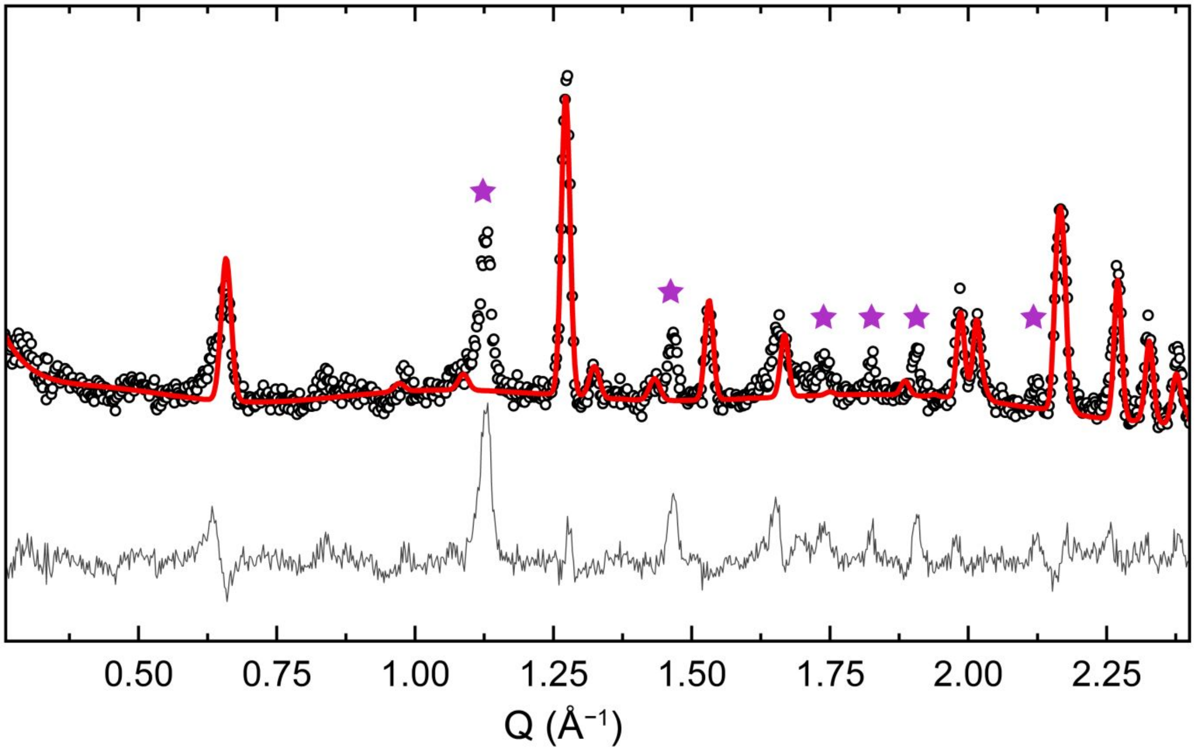

Figure S2 | By eye, we identified six apparent, well isolated magnetic Bragg peaks in the pattern collected for 12R-BCM at 4.2 K, which are denoted by purple stars here. The black circles, red curve, and grey curve denote the raw data, the Rietveld refinement curve, and the difference curve, respectively.

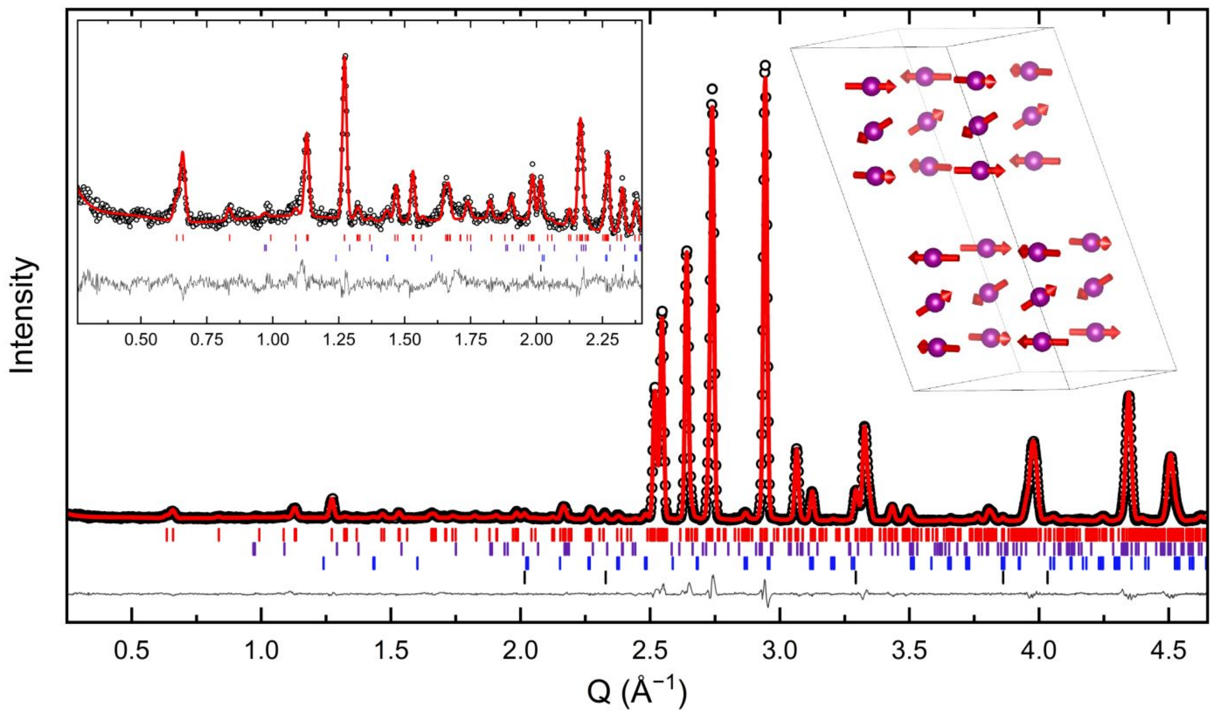

Figure S3 | Rietveld refinement of the PND pattern obtained for 12R-BCM. T = 4.2 K, λ = 2.4062 Å, R_{wp} = 4.573 %, R_p = 3.527 %, R_{exp} = 2.326 %, GOF = 1.966. The black circles, red curve, and grey curve denote the raw data, the Rietveld refinement curve, and the difference curve, respectively. The vertical red, purple, blue, and black tick marks denote the *hkl* positions for the 12R-BCM nuclear and magnetic phases combined, $Ba_4Mn_3O_{12}$, $BaCeO_3$, and CeO_2, respectively. Symbols are larger than or commensurate with their error bars which represent $\pm1\sigma$. The inset (left) illustrates the data and fit in the low-Q region of the pattern. The magnetic contributions to the pattern are now well fitted. The inset (right) displays one unit cell of the resulting magnetic structure for irrep. Γ_1, magnetic space group 15.91. Purple spheres depict Mn^{4+} ions, and red arrows depict refined magnetic moments.

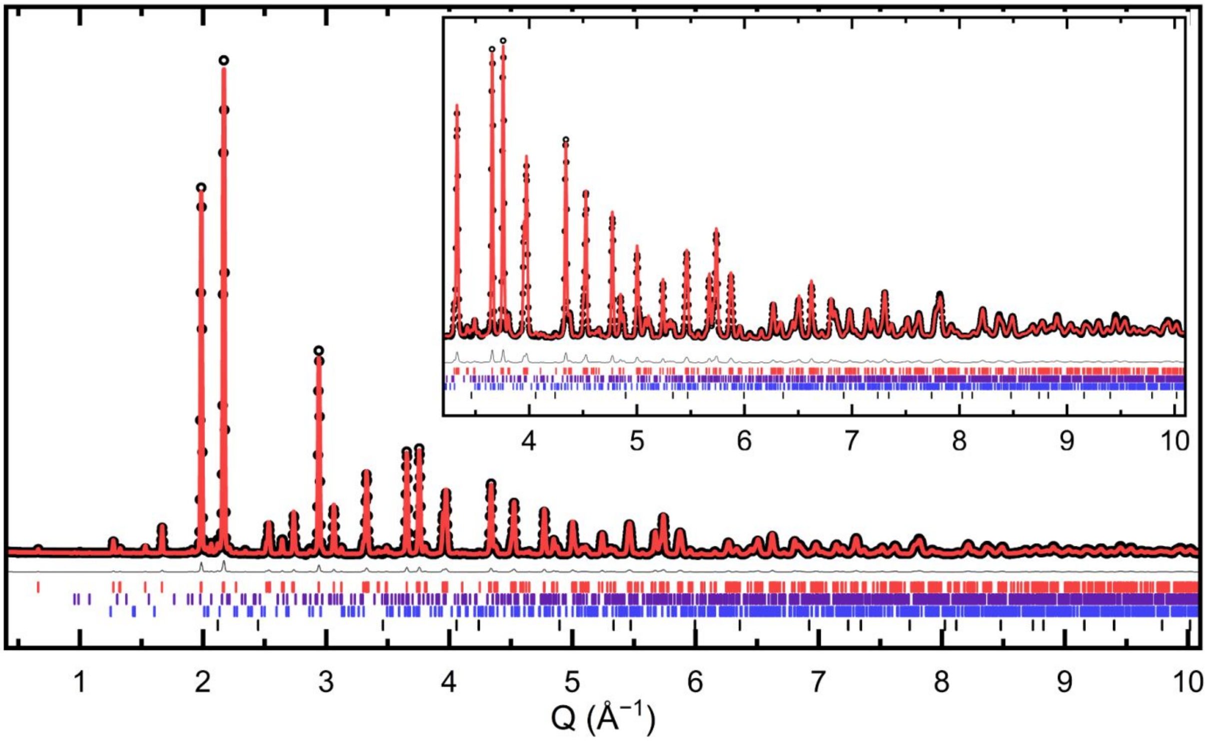

Figure S4 | Rietveld refinement of the SPXRD pattern obtained for 12R-BPM. T = 100 K, λ = 0.1821 Å, R_{wp} = 4.502 %, R_p = 3.246 %, R_{exp} = 0.932 %, GOF = 4.830. The black circles, red curve, and grey curve denote the raw data, the Rietveld refinement curve, and the difference curve, respectively. The vertical red, purple, blue and black tick marks denote the *hkl* positions for 12R-BPM, $Ba_4Mn_3O_{12}$, $BaPrO_3$, and a minor PrO_2 impurity phase, respectively. Symbols are larger than or commensurate with their error bars which represent ±1σ. The inset illustrates the data and fit in the high-Q region of the pattern.

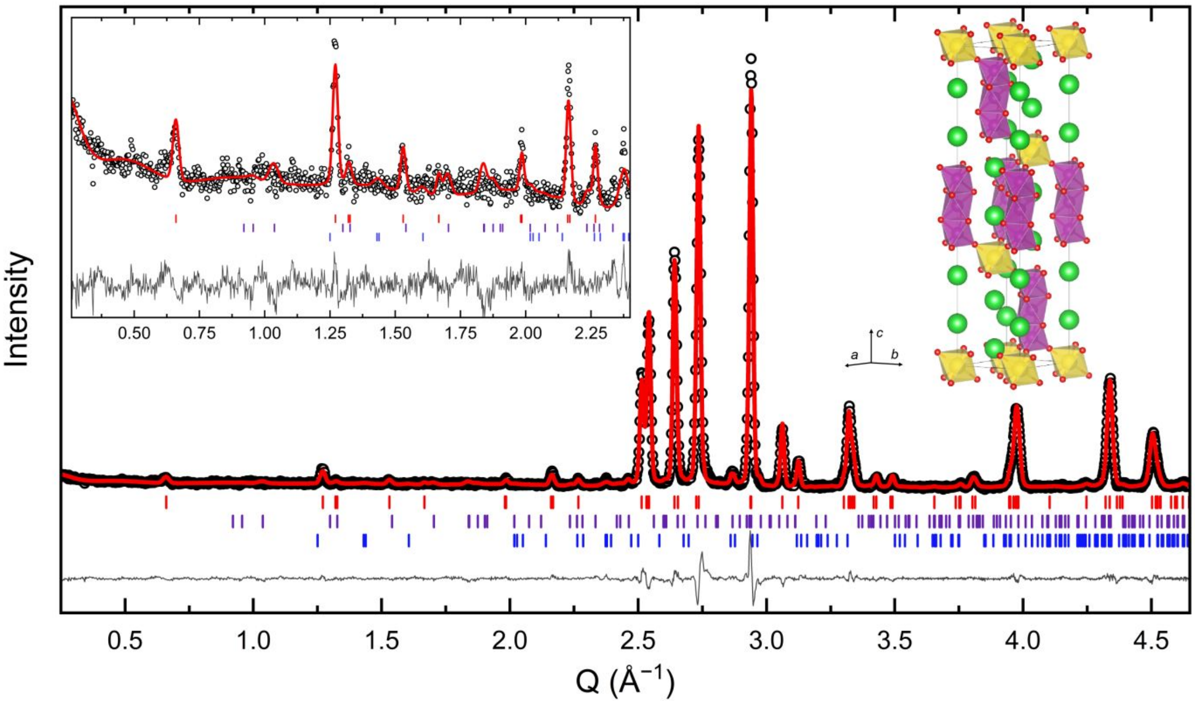

Figure S5 | Rietveld refinement of the PND pattern obtained for 12R-BPM. T = 250 K, λ = 2.4062 Å, R_{wp} = 6.367 %, R_p = 4.784 %, R_{exp} = 3.163 %, GOF = 1.951. The black circles, red curve, and grey curve denote the raw data, the Rietveld refinement curve, and the difference curve, respectively. The vertical red, purple, and blue tick marks denote the *hkl* positions for the 12R-BPM, $Ba_4Mn_3O_{12}$, and $BaPrO_3$ phases, respectively. Symbols are larger than or commensurate with their error bars which represent ±1σ. The inset (left) illustrates the data and fit in the low-Q region of the pattern. The magnetic contributions to the pattern are now well fitted. The inset (right) displays one unit cell of the resulting crystal structure. Purple and gold polyhedra depict Mn and Pr coordination environments, while green and red spheres depict Ba and O atoms, respectively.

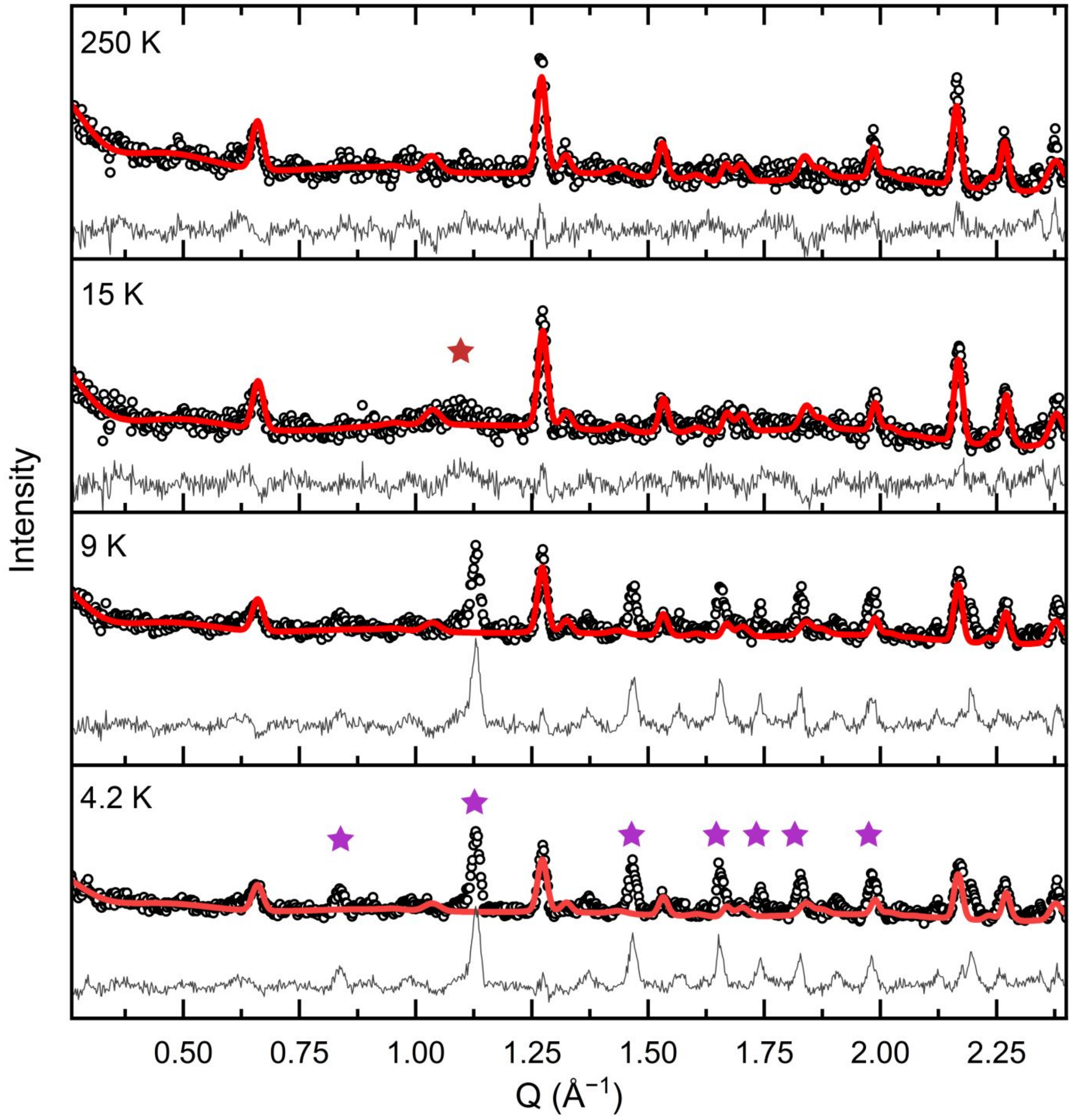

Figure S6 | Plots of PND patterns collected for 12R-BPM at 250 K, 15 K, 9 K, and 4.2 K. The nuclear-only model obtained for the data collected at 250 K was applied to all four data sets, and difference curves were generated to highlight possible magnetic scattering contributions to the patterns. At 15 K, by eye, the only apparent change is a new broad, low-intensity feature at $Q \approx 1.1$ Å^{-1}, denoted by an orange star above the pattern. At 9 K and 4.2 K, we observe by eye new Bragg peaks which are not captured by the nuclear phase-only model. They are sharp and intense, and we tentatively attribute these as magnetic Bragg peaks. The new Bragg peaks which are well isolated in the 4.2 K pattern, indicated by purple stars, were used to index the magnetic propagation vector. The black circles, red curve, and grey curve denote the raw data, the Rietveld refinement curve, and the difference curve, respectively.

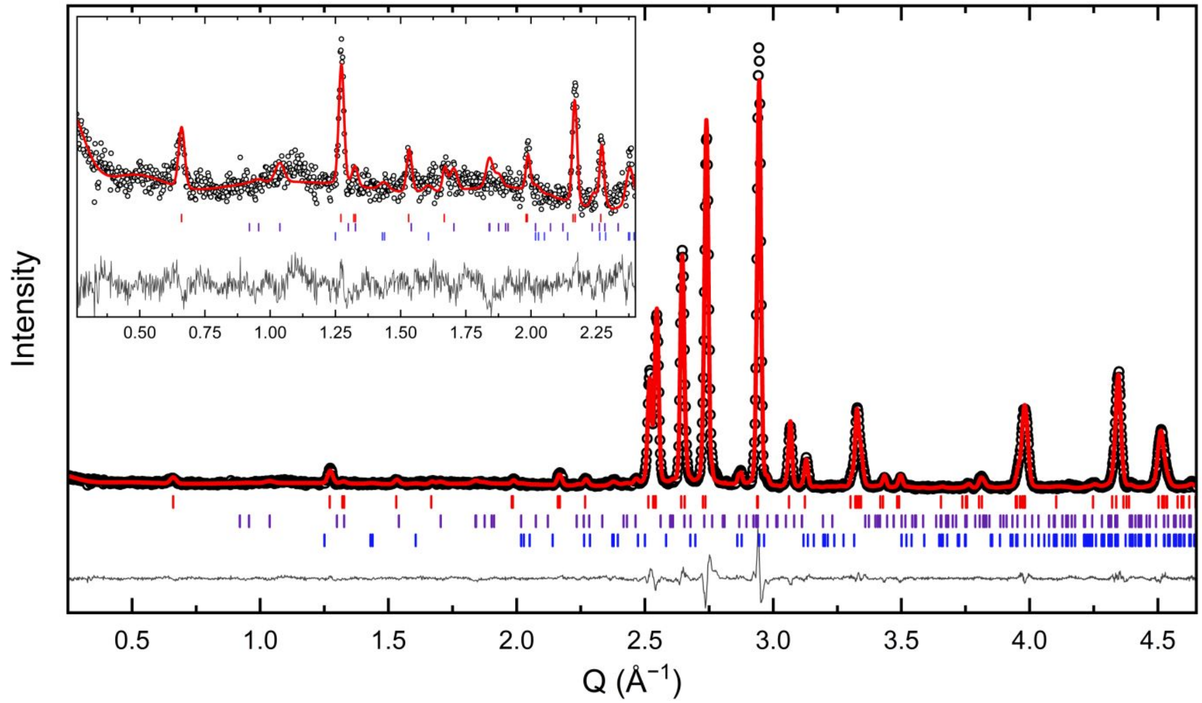

Figure S7 | Rietveld refinement of the PND pattern obtained for 12R-BPM. T = 15 K, λ = 2.4062 Å, R_{wp} = 6.658 %, R_p = 5.068 %, R_{exp} = 3.250 %, GOF = 2.049. The black circles, red curve, and grey curve denote the raw data, the Rietveld refinement curve, and the difference curve, respectively. The vertical red, purple, and blue tick marks denote the *hkl* positions for the 12R-BPM, $Ba_4Mn_3O_{12}$, and $BaPrO_3$ phases, respectively. Symbols are larger than or commensurate with their error bars which represent $\pm 1\sigma$. The inset (left) illustrates the data and fit in the low-Q region of the pattern. The magnetic contributions to the pattern are now well fitted.

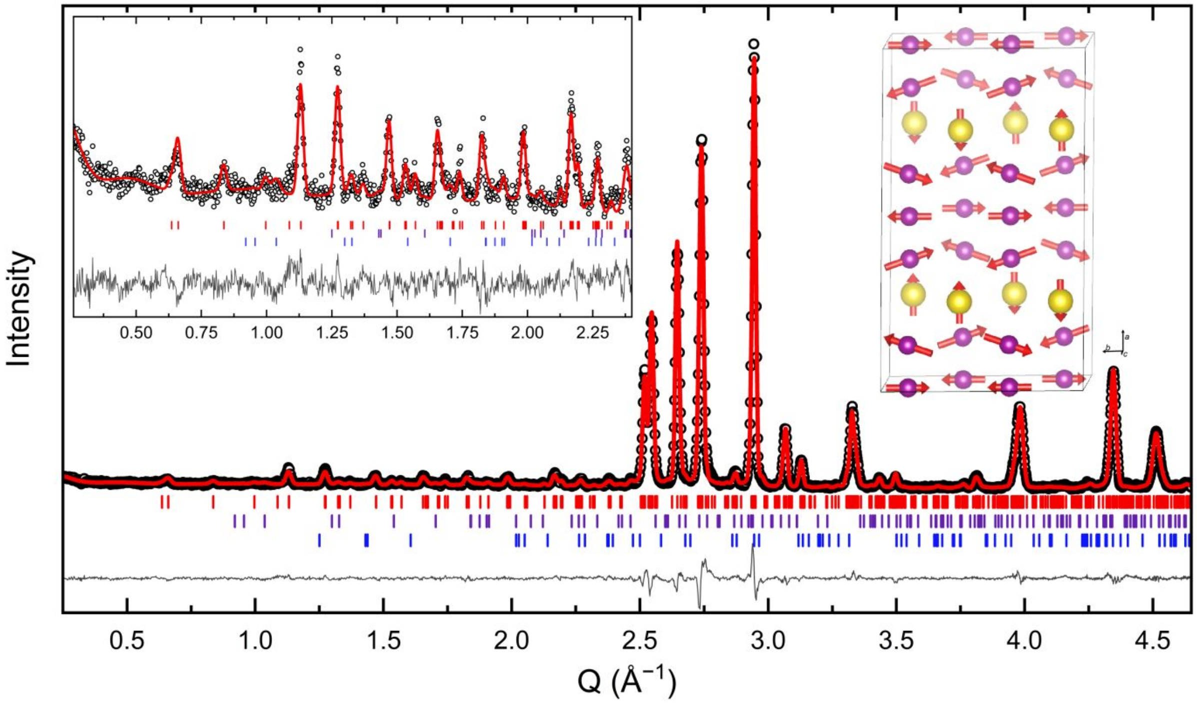

Figure S8 | Rietveld refinement of the PND pattern obtained for 12R-BPM. $T = 9$ K, $\lambda = 2.4062$ Å, $R_{wp} = 6.468$ %, $R_p = 4.954$ %, $R_{exp} = 3.223$ %, GOF = 2.007. The black circles, red curve, and grey curve denote the raw data, the Rietveld refinement curve, and the difference curve, respectively. The vertical red, purple, and blue tick marks denote the *hkl* positions for the 12R-BPM nuclear and magnetic phases combined, $Ba_4Mn_3O_{12}$, and $BaPrO_3$ phases, respectively. Symbols are larger than or commensurate with their error bars which represent ±1σ. The inset (left) illustrates the data and fit in the low-*Q* region of the pattern. The magnetic contributions to the pattern are now well fitted. The inset (right) displays one unit cell of the resulting magnetic structure for irrep. Γ_2, magnetic space group 15.91. Purple and gold spheres depict Mn^{4+} and Pr^{4+} ions, while red arrows depict refined magnetic moments.

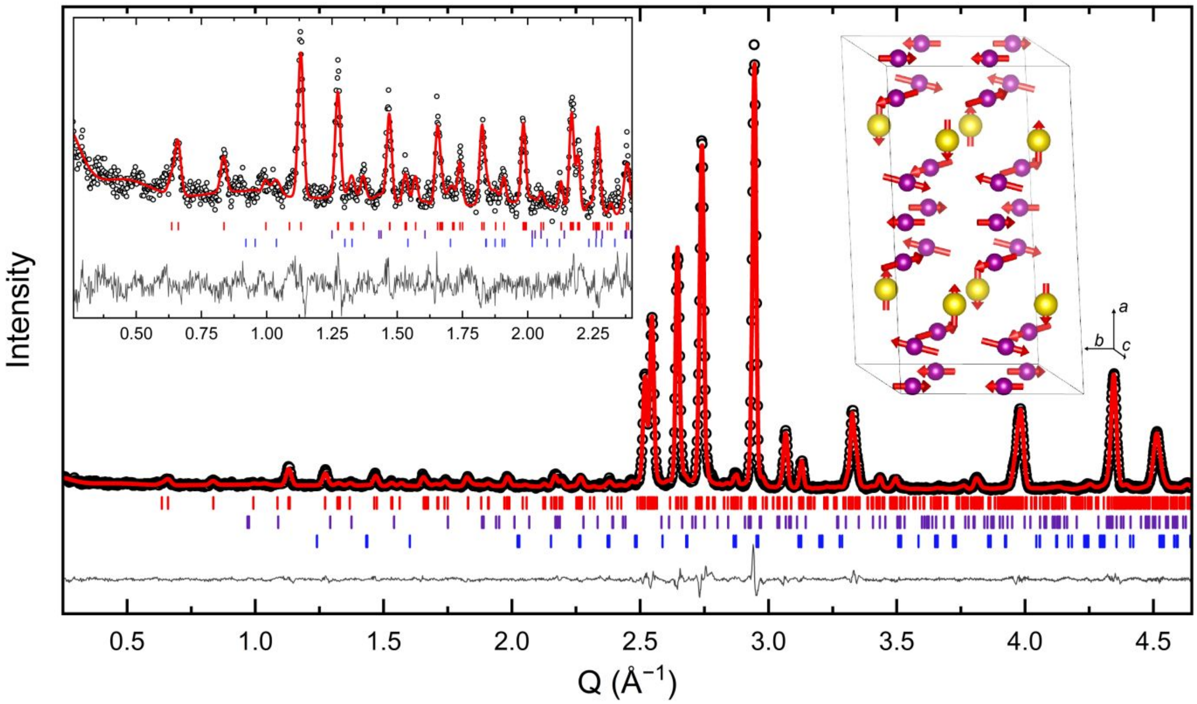

Figure S9 | Rietveld refinement of the PND pattern obtained for 12R-BPM. T = 4.2 K, λ = 2.4062 Å, R_{wp} = 5.755 %, R_p = 4.415 %, R_{exp} = 3.249 %, GOF = 1.772. The black circles, red curve, and grey curve denote the raw data, the Rietveld refinement curve, and the difference curve, respectively. The vertical red, purple, and blue tick marks denote the *hkl* positions for the 12R-BPM nuclear and magnetic phases combined, $Ba_4Mn_3O_{12}$, and $BaPrO_3$, respectively. Symbols are larger than or commensurate with their error bars which represent $\pm 1\sigma$. The inset (left) illustrates the data and fit in the low-Q region of the pattern. The magnetic contributions to the pattern are now well fitted. The inset (right) displays one unit cell of the resulting magnetic structure for irrep. Γ_2, magnetic space group 15.91. Purple and gold spheres depict Mn^{4+} and Pr^{4+} ions, while red arrows depict refined magnetic moments.

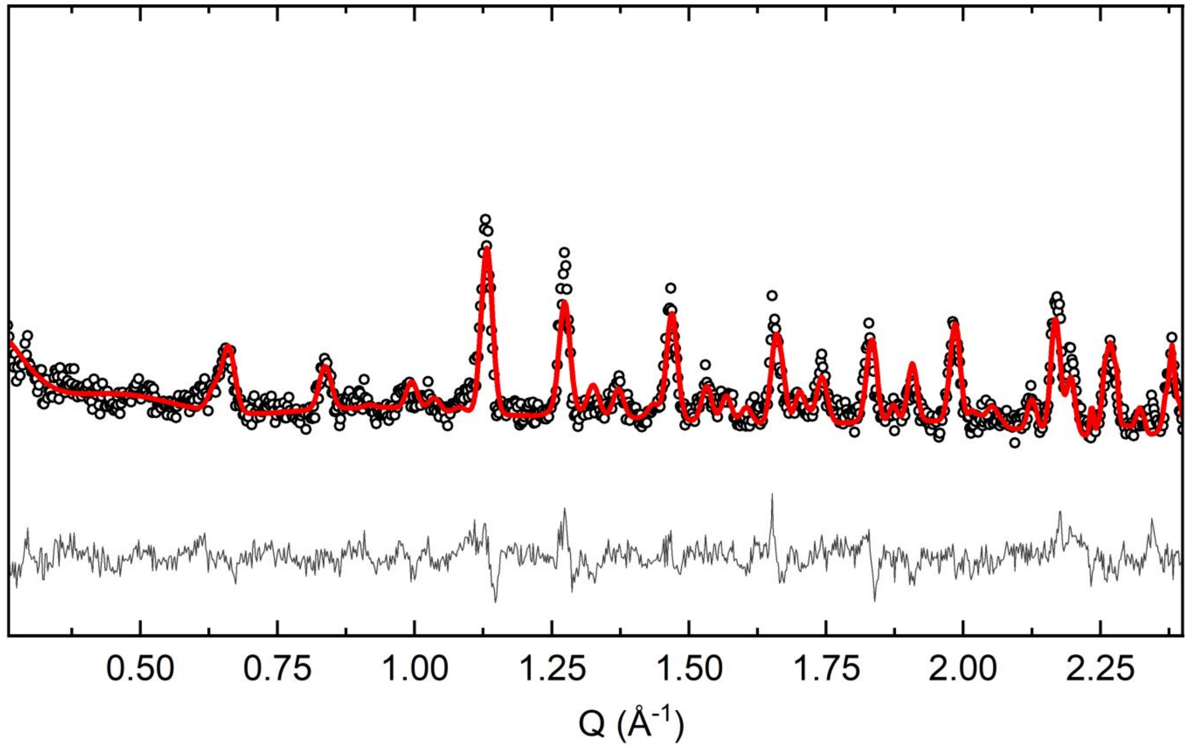

Figure S10 | The fit to the magnetic Bragg peaks using magnetic structure Γ_1, magnetic space group 5.17 is slightly worse than the best fit both visually and by the fit statistic, as given in Table 1 in the main text.

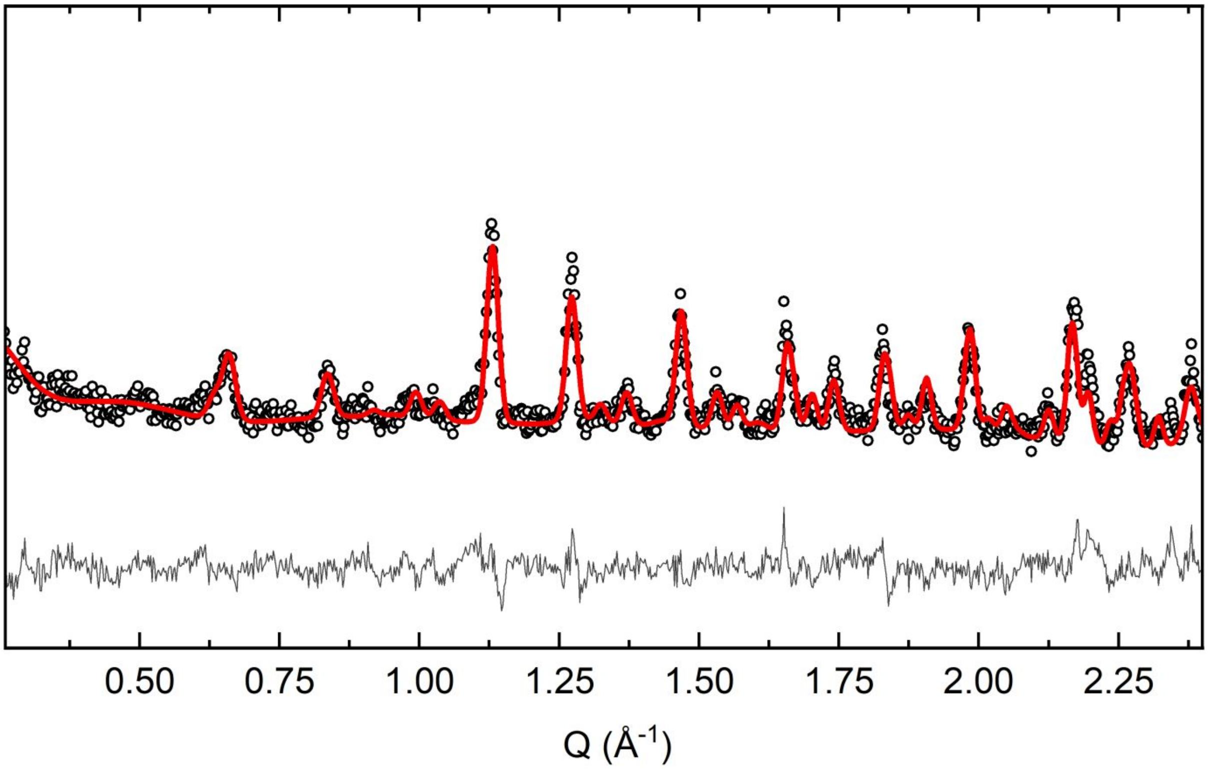

Figure S11 | The fit to the magnetic Bragg peaks using magnetic structure Γ_2, magnetic space group 5.17 is slightly worse than the best fit both visually and by the fit statistic, as given in Table 1 in the main text.

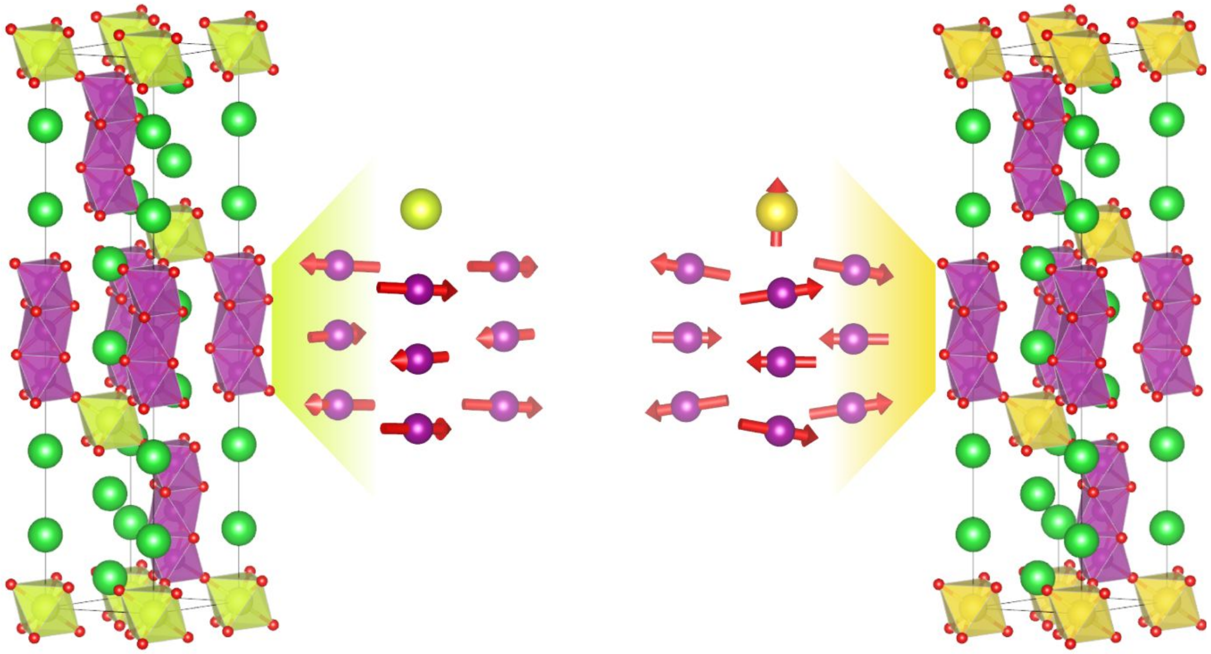

Figure S12 | The magnetic and nuclear crystal structures for 12R-BCM and 12R-BPM are shown. (Left) 12R-BCM contains Mn_3O_{12} trimers that display strong intratrimer coupling but weak or negligible intertrimer coupling, thanks in part to the diamagnetic Ba^{2+} and Ce^{4+} ions create poor coupling pathways. The magnetic moments are only modestly canted out of plane. (Right) In 12R-BPM, the Mn_3O_{12} trimers are bridged by magnetic Pr^{4+} ions, which creates a stronger intratrimer coupling pathway, and the spins are canted out of the plane.

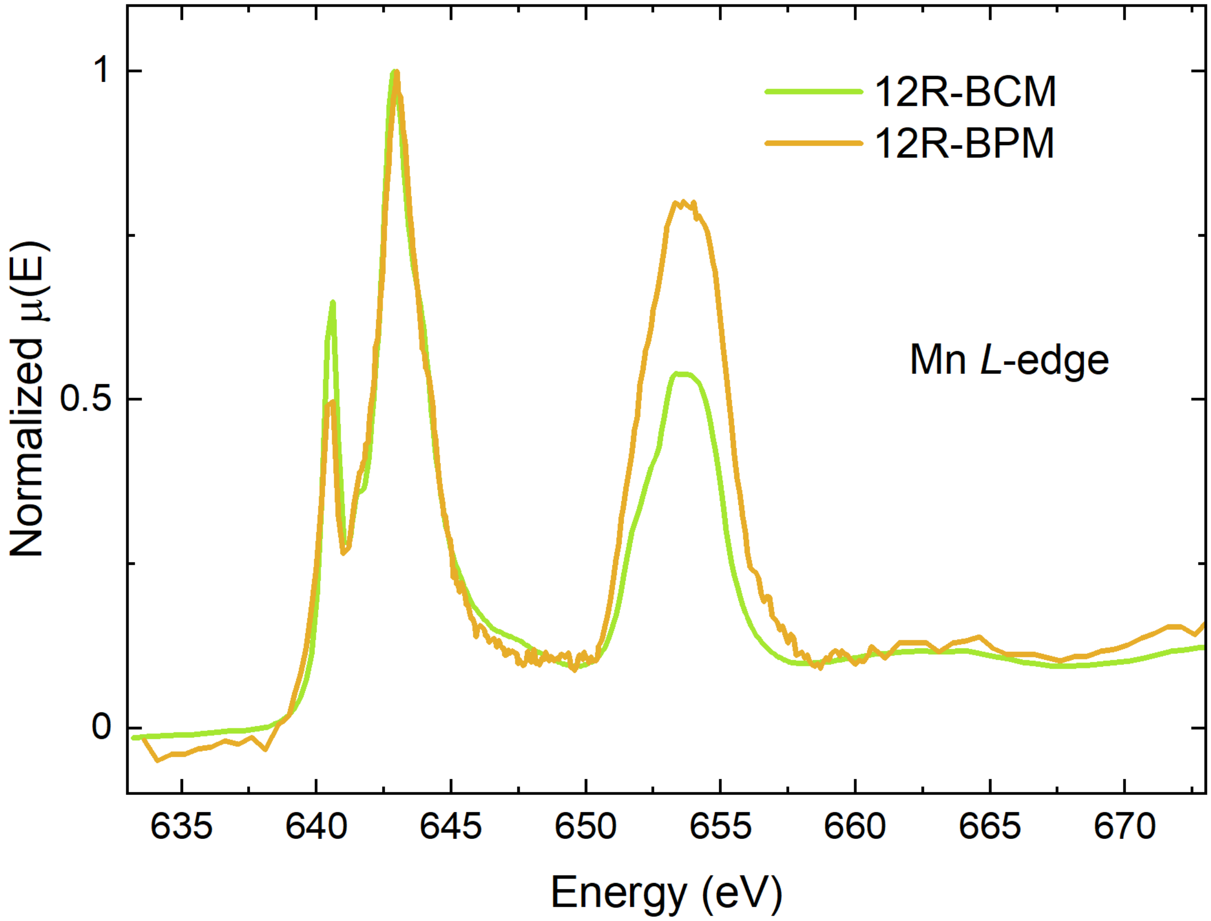

Figure S13 | Background-corrected and normalized XAS spectra at the Mn *L*-edge for both 12R-BCM and 12R-BPM.

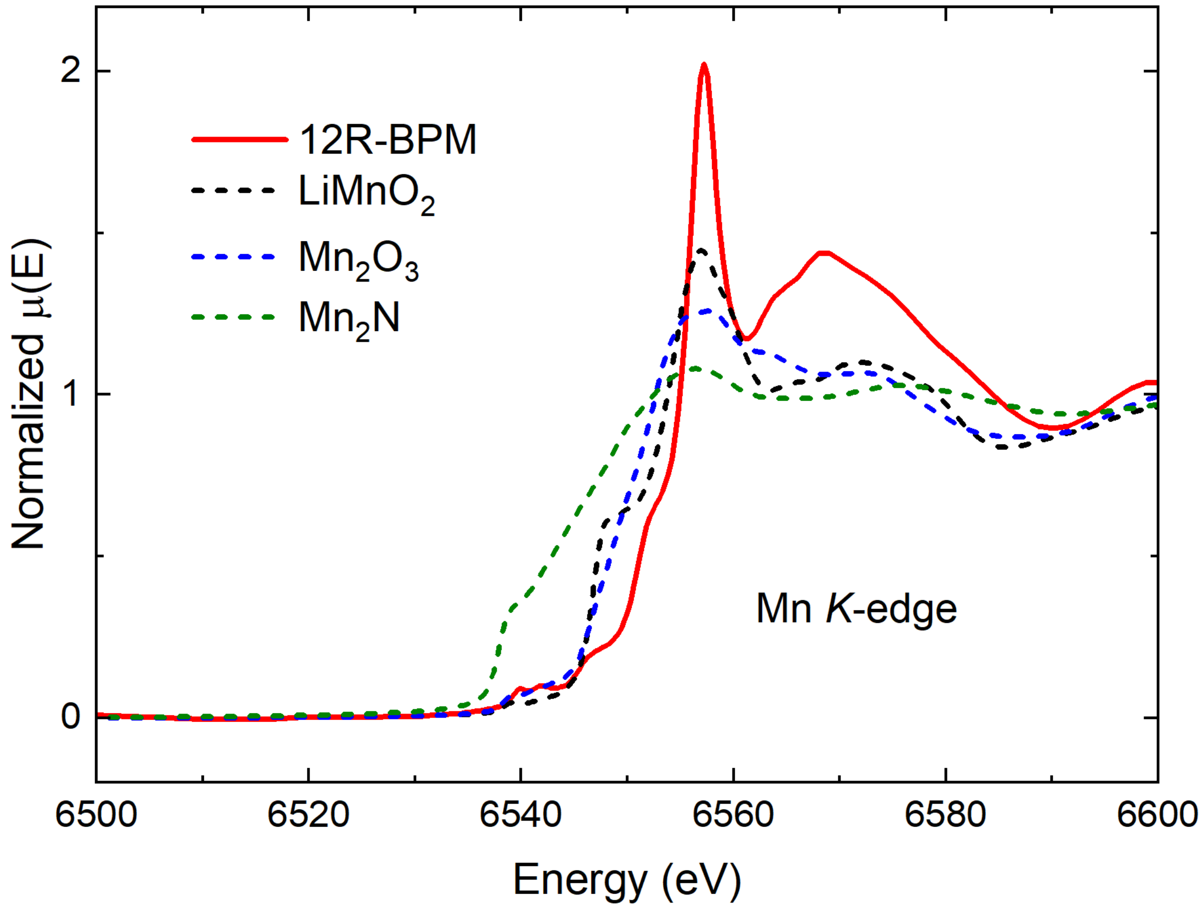

Figure S14 | Normalized XAS spectra at the Mn *K*-edge for 12R-BCM and $LiMnO_2$, Mn_2O_3, and Mn_2N standards.

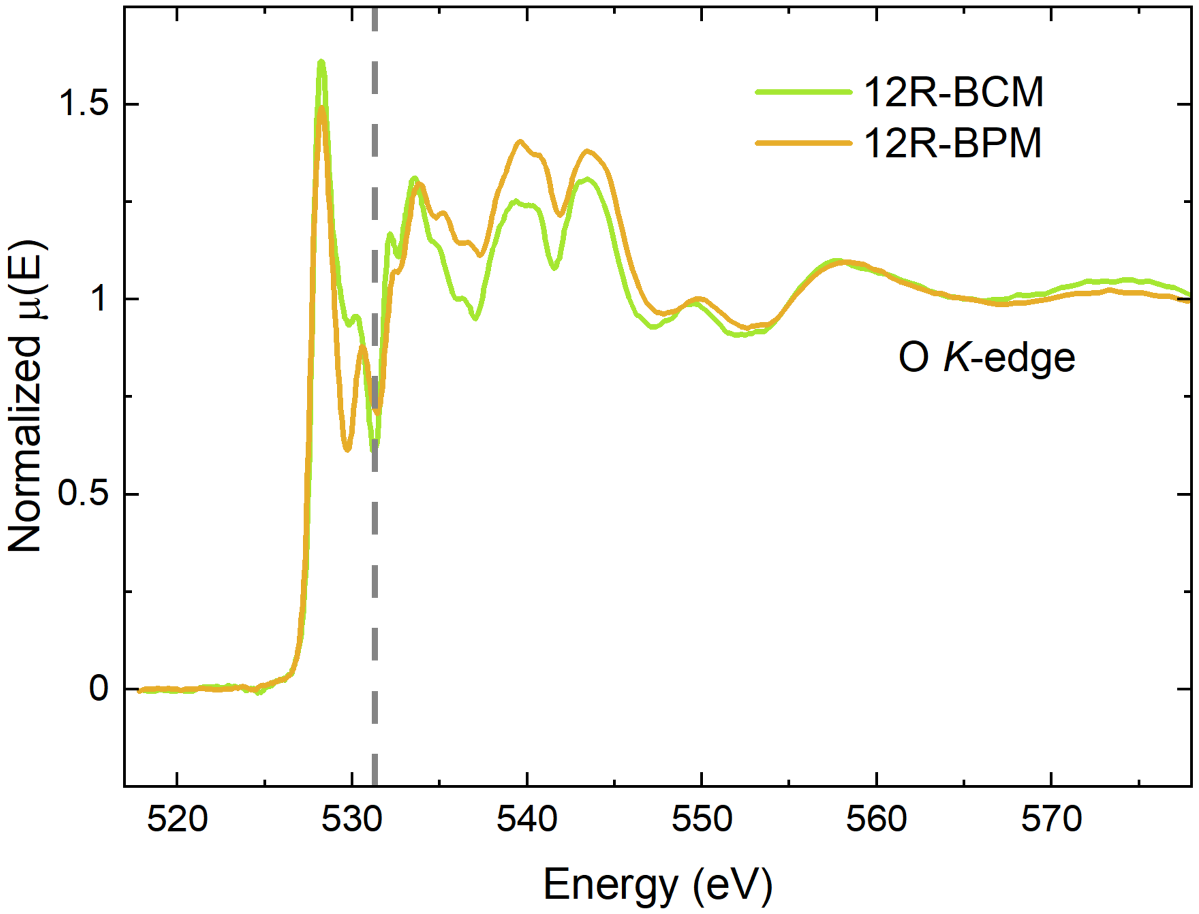

Figure S15 | Background-corrected and normalized XAS spectra at the O K-edge for both 12R-BCM and 12R-BPM, annotated with a dashed line to delineate the different O transition regimes.

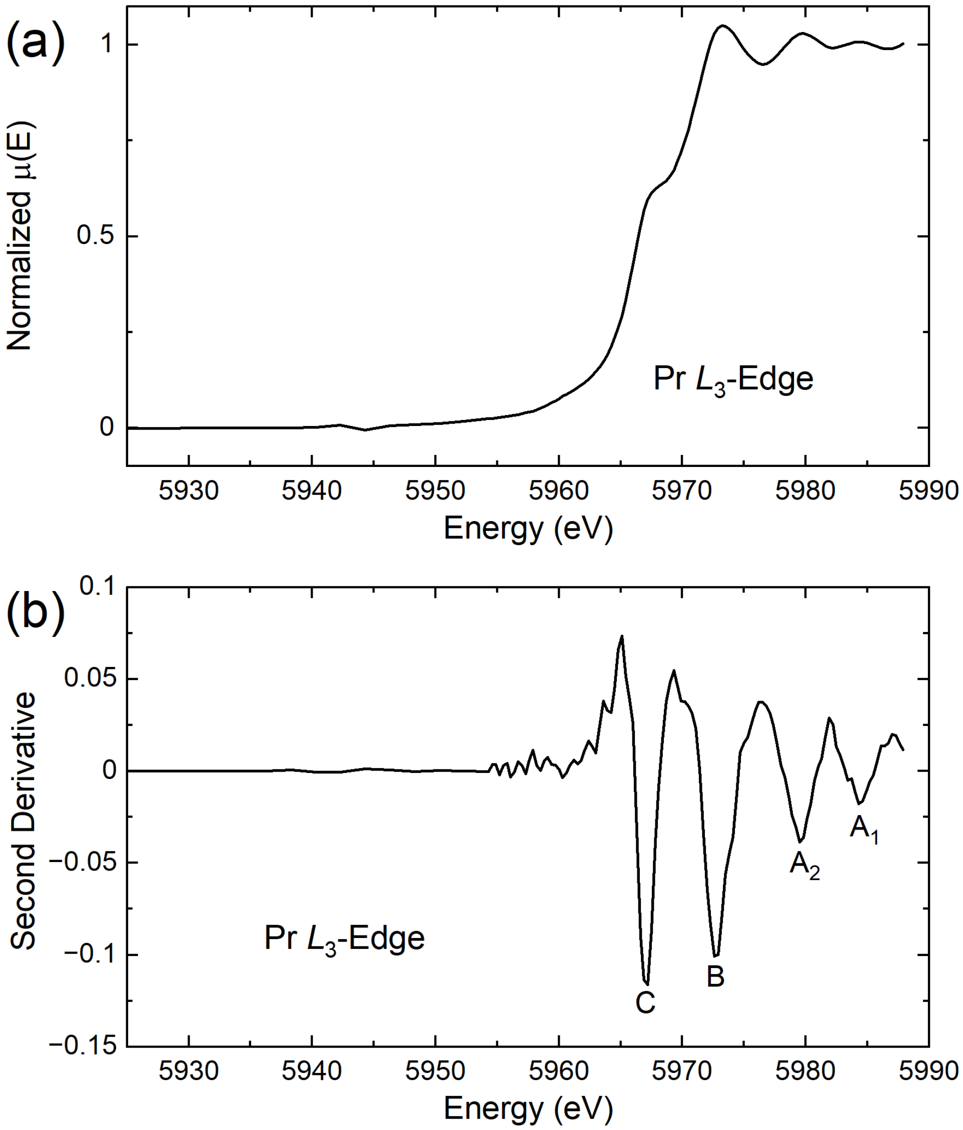

Figure S16 | a) Normalized XAS spectrum and (b) second derivative of the XAS spectrum at the Pr *L*-edge for 12R-BPM.

IR	Mag. SG	Ion	$m_{\parallel a}$ (μ_B)	$m_{\parallel b}$ (μ_B)	$m_{\parallel c}$ (μ_B)	Moment (μ_B)	Moment per f.u. (μ_B)
Γ_1	15.91	Mn_1	−0.2(2)	0	−1.1(1)	1.1(2)	2.1(2)
		Mn_2	−0.48(8)	−0.25(9)	1.10(9)	1.2(1)	

Table S1 | Refined magnetic moments for 12R-BCM at 4.2 K. IR and Mag S.G. stand for irreducible representation and magnetic space group, respectively. Moments are defined with respect to magnetic unit cell axes, which differ for the two systems (see mcifs for further details).

IR	Mag. SG	Ion	$m_{\parallel a}$ (μ_B)	$m_{\parallel b}$ (μ_B)	$m_{\parallel c}$ (μ_B)	Moment (μ_B)	Moment per f.u. (μ_B)
		Mn_1	0	−1.72(7)	0	−1.72(7)	
Γ_2	15.91	Mn_2	0.5(1)	−1.99(4)	−0.44(9)	2.1(2)	2.7(2)
		Pr	−0.4(1)	0	−0.13(5)	0.4(1)	

Table S2 | Refined magnetic moments for 12R-BPM at 4.2 K. IR and Mag S.G. stand for irreducible representation and magnetic space group, respectively. Moments are defined with respect to magnetic unit cell axes, which differ for the two systems (see mcifs for further details).

IR	Mag. SG	Ion	$m_{\parallel a}$ (μ_B)	$m_{\parallel b}$ (μ_B)	$m_{\parallel c}$ (μ_B)	Moment (μ_B)	Moment per f.u. (μ_B)
		Mn_1	0	−1.60(8)	0	1.60(8)	
Γ_2	15.91	Mn_2	0.6(1)	−1.75(5)	−0.2(1)	1.9(2)	2.5(3)
		Pr	−0.4(1)	0	−0.12(5)	0.4(2)	

Table S3 | Refined magnetic moments for 12R-BPM at 9 K. IR and Mag S.G. stand for irreducible representation and magnetic space group, respectively. Moments are defined with respect to magnetic unit cell axes, which differ for the two systems (see mcifs for further details).

12R-BCM	Source	HB-2A<br>HFIR<br>ORNL	Crystal System	Hexagonal
	Temperature (K)	15	Space Group	$R\bar{3}m$
	Wavelength (Å)	2.4062	a (Å)	5.78306(3)
	Formula	$Ba_4CeMn_3O_{12}$	c (Å)	28.5370(3)
	Weight (g mol⁻¹)	1,046.26	Volume (Å³)	826.522(12)
	Q range (Å⁻¹)	0.2–4.7	Z	1
	R_{wp} (%)	4.267	R_p (%)	3.361
	R_{exp} (%)	2.365	GOF	1.804

Table S4 | Crystallographic data for 12R-BCM derived from Rietveld refinement of the PND pattern collected at 15 K. Numbers in parentheses indicate ±1σ. Listed *R* values are as defined in TOPAS.[2]

	Multiplicity	x	y	z	Occupancy	B_{iso}
Ba1	6	0	0	0.13140(10)	1	0.10(7)
Ba2	6	0	0	0.28255(9)	1	0.22(8)
Pr1	3	0	0	0	1	1.38(12)
Mn1	3	0	0	0.5	1	1.03(17)
Mn2	6	0	0	0.41319(14)	1	0.10(10)
O1	18	0.18594(14)	0.81406(14)	0.20877(5)	1	0.25(3)
O2	18	0.48983(12)	0.51017(12)	0.28894(5)	1	0.50(3)

Table S5 | Crystallographic data for 12R-BCM derived from Rietveld refinement of the PND pattern collected at 15 K. Numbers in parentheses indicate ±1σ.

12R-BCM	Source	HB-2A<br>HFIR<br>ORNL	Crystal System	Monoclinic
	Temperature (K)	4.2	Space Group	15.91
	Wavelength (Å)	2.4062	*a* (Å)	20.1806(4)
	Formula	$Ba_4CeMn_3O_{12}$	*b* (Å)	11.5704(4)
	Weight (g mol^{-1})	1,046.26	*c* (Å)	10.0176(4)
	Q range (Å^{-1})	0.2–4.7	β (°)	109.448(3)
	R_{wp} (%)	4.573	Volume (Å^3)	2205.7(2)
	R_{exp} (%)	2.326	Z	1
	R_p (%)	3.527	GOF	1.966

Table S6 | Crystallographic data for 12R-BCM derived from Rietveld refinement of the PND pattern collected at 4.2 K. Numbers in parentheses indicate ±1σ. Listed *R* values are as defined in TOPAS.[2]

	Multiplicity	x	y	z	Occupancy	B_{iso}
Ba1_1	16	0.80253(17)	0.375	0.618(2)	1	0.10(8)
Ba2_1	16	0.57640(16)	0.375	0.468(2)	1	0.85(10)
Ce1_1	8	0	0.625	0.25	1	1.71(16)
Mn1_1	8	0.25	0.375	0.25	1	1.7(2)
Mn2_1	16	0.3798(2)	0.375	0.337(2)	1	0.47(11)
O1_1	16	0.6867(8)	0.375	0.3550(16)	1	0.4(4)
O1_2	16	0.6867(4)	0.2354(9)	0.6342(10)	1	0.28(19)
O1_3	16	0.6867(4)	0.4854(9)	0.1342(10)	1	0.28(19)
O2_1	16	0.5668(8)	0.375	−0.0281(18)	1	1.9(3)
O2_2	16	0.5668(4)	0.0080(10)	0.7059(12)	1	0.15(12)
O2_3	16	0.5668(4)	0.2580(10)	0.2059(12)	1	0.15(12)

Table S7 | Crystallographic data for 12R-BCM derived from Rietveld refinement of the PND pattern collected at 4.2 K. Numbers in parentheses indicate ±1σ.

	Multiplicity	x	y	z	Occupancy	B_{iso}
Ba1	6	0	0	0.13125(6)	1	0.29(3)
Ba2	6	0	0	0.28262(5)	1	0.19(3)
Pr1	3	0	0	0	1	0.21(4)
Mn1	3	0	0	0.5	1	0.50(10
Mn2	6	0	0	0.41396(13)	1	0.10(9)
O1	18	0.1892(7)	0.8108(7)	0.2081(3)	1	0.54(17)
O2	18	0.4934(8)	0.5066(8)	0.2887(3)	1	0.30(15)

Table S8 | Crystallographic data for 12R-BPM derived from Rietveld refinement of the SPXRD pattern collected at 100 K. Numbers in parentheses indicate ±1σ.

12R-BPM	Source	28-ID-II NSLS II BNL	Crystal System	Hexagonal
	Temperature (K)	100	Space Group	$R\bar{3}m$
	Wavelength (Å)	0.1821	a (Å)	5.79184(4)
	Formula	$Ba_4PrMn_3O_{12}$	c (Å)	28.5362(4)
	Weight (g mol^{-1})	1,046.26	Volume (Å^3)	829.010(16)
	Q range (Å^{-1})	0.3–10.2	Z	1
	R_{wp} (%)	4.502	R_p (%)	3.246
	R_{exp} (%)	0.932	GOF	4.830

Table S9 | Crystallographic data for 12R-BPM derived from Rietveld refinement of the SPXRD pattern collected at 100 K. Numbers in parentheses indicate ±1σ. Listed *R* values are as defined in TOPAS.[2]

**12R-BPM**	**Source**	HB-2A<br>HFIR<br>ORNL	**Crystal System**	Hexagonal
	**Temperature (K)**	250	**Space Group**	$R\overline{3}m$
	**Wavelength (Å)**	2.4062	***a*** **(Å)**	5.79167(8)
	**Formula**	$Ba_4PrMn_3O_{12}$	***c*** **(Å)**	28.5264(6)
	**Weight (g mol⁻¹)**	1,047.05	**Volume (Å³)**	828.68(3)
	**Q range (Å⁻¹)**	0.2–4.7	**Z**	1
	**R_{wp} (%)**	6.367	**R_p (%)**	4.784
	**R_{exp} (%)**	3.163	**GOF**	1.951

Table S10 | Crystallographic data for 12R-BPM derived from Rietveld refinement of the PND pattern collected at 250 K. Numbers in parentheses indicate ±1σ. Listed *R* values are as defined in TOPAS.[2]

	**Multipliciy**	**x**	**y**	**z**	**Occupancy**	**B_{iso}**
Ba1	6	0	0	0.1321(2)	1	0.50(17)
Ba2	6	0	0	0.28300(19)	1	0.5(2)
Pr1	3	0	0	0	1	0.5(3)
Mn1	3	0	0	0.5	1	3.0(4)
Mn2	6	0	0	0.4144(4)	1	2.0(2)
O1	18	0.1870(3)	0.8130(3)	0.20853(11)	1	0.77(8)
O2	18	0.4896(3)	0.5104(3)	0.28893(11)	1	1.11(7)

Table S11 | Crystallographic data for 12R-BPM derived from Rietveld refinement of the PND pattern collected at 250 K. Numbers in parentheses indicate ±1σ.

**12R-BPM**	**Source**	HB-2A HFIR ORNL	**Crystal System**	Hexagonal
	**Temperature (K)**	15	**Space Group**	$R\bar{3}m$
	**Wavelength (Å)**	2.4062	***a*** **(Å)**	5.78199(7)
	**Formula**	$Ba_4PrMn_3O_{12}$	***c*** **(Å)**	28.4884(7)
	**Weight (g mol⁻¹)**	1,047.05	**Volume (Å³)**	824.81(3)
	**Q range (Å⁻¹)**	0.2–4.7	**Z**	1
	**R_{wp} (%)**	6.658	**R_p (%)**	5.068
	**R_{exp} (%)**	3.250	**GOF**	2.049

Table S13 | Crystallographic data for 12R-BPM derived from Rietveld refinement of the PND pattern collected at 15 K. Numbers in parentheses indicate ±1σ. Listed *R* values are as defined in TOPAS.[2]

	**Multipliciy**	**x**	**y**	**z**	**Occupancy**	B_{iso}
Ba1	6	0	0	0.1321(2)	1	0.50(17)
Ba2	6	0	0	0.28293(19)	1	0.5(2)
Pr1	3	0	0	0	1	0.5(3)
Mn1	3	0	0	0.5	1	2.5(4)
Mn2	6	0	0	0.4146(4)	1	1.8(2)
O1	18	0.1867(3)	0.8133(3)	0.20855(11)	1	0.53(8)
O2	18	0.4895(3)	0.5105(3)	0.28885(11)	1	0.80(7)

Table S14 | Crystallographic data for 12R-BPM derived from Rietveld refinement of the PND pattern collected at 15 K. Numbers in parentheses indicate ±1σ.

12R-BPM	Source	HB-2A<br>HFIR<br>ORNL	Crystal System	Monoclinic
	Temperature (K)	9	Space Group	15.91
	Wavelength (Å)	2.4062	*a* (Å)	20.1499(6)
	Formula	$Ba_4CeMn_3O_{12}$	*b* (Å)	11.5532(5)
	Weight (g mol^{-1})	1,046.26	*c* (Å)	10.0241(4)
	Q range (Å^{-1})	0.2–4.7	β (°)	109.532(4)
	R_{wp} (%)	6.468	Volume (Å^3)	2199.3(2)
	R_{exp} (%)	3.223	Z	1
	R_p (%)	4.954	GOF	2.007

Table S15 | Crystallographic data for 12R-BPM derived from Rietveld refinement of the PND pattern collected at 9 K. Numbers in parentheses indicate ±1σ. Listed *R* values are as defined in TOPAS.[2]

	Multipliciy	x	y	z	Occupancy	B_{iso}
Ba1_1	16	0.0530(4)	0.125	0.6235(18)	1	0.50(18)
Ba2_1	16	0.8238(4)	0.125	0.4600(18)	1	1.6(2)
Ce1_1	8	0.25	0.375	0.25	1	0.5(3)
Mn1_1	8	0	0.125	0.25	1	3.6(4)
Mn2_1	16	0.6293(5)	0.125	0.329(2)	1	2.8(3)
O1_1	16	0.9338(8)	0.125	0.3512(15)	1	0.9(6)
O1_2	16	0.9410(9)	0.4854(15)	0.6269(13)	1	0.5(5)
O1_3	16	0.9370(9)	0.2419(16)	0.1412(17)	1	0.5(4)
O2_1	16	0.8141(8)	0.125	−0.0337(14)	1	0.5(4)
O2_2	16	0.8153(10)	0.260(2)	0.707(2)	1	0.5(4)
O2_3	16	0.8219(9)	0.010(2)	0.1996(19)	1	1.0(5)

Table S16 | Crystallographic data for the nuclear phase of 12R-BPM derived from the Rietveld refinement of the PND pattern collected at 9 K. Numbers in parentheses indicate ±1σ. The somewhat large B_{iso} values of approximately 3 for the Mn ions may be an artifact of the limited *Q* range of the data set. The model is consistent with the data. Additional measurements with expanded *Q* ranges may help constrain these values.

12R-BPM	Source	HB-2A HFIR ORNL	Crystal System	Monoclinic
	Temperature (K)	4.2	Space Group	15.91
	Wavelength (Å)	2.4062	*a* (Å)	20.1529(7)
	Formula	$Ba_4CeMn_3O_{12}$	*b* (Å)	11.5557(5)
	Weight (g mol^{-1})	1,046.26	*c* (Å)	10.0253(5)
	Q range (Å^{-1})	0.2–4.7	β (°)	109.522(5)
	R_{wp} (%)	5.755	Volume (Å^3)	2200.5(2)
	R_{exp} (%)	3.249	Z	1
	R_p (%)	4.415	GOF	1.772

Table S17 | Crystallographic data for 12R-BPM derived from Rietveld refinement of the PND pattern collected at 4.2 K. Numbers in parentheses indicate ±1σ. Listed *R* values are as defined in TOPAS.[2]

	Multipliciy	x	y	z	Occupancy	B_{iso}
Ba1_1	16	0.0520(4)	0.125	0.618(3)	1	0.50(17)
Ba2_1	16	0.8252(3)	0.125	0.467(2)	1	0.5(2)
Ce1_1	8	0.25	0.375	0.25	1	1.2(3)
Mn1_1	8	0	0.125	0.25	1	3.3(4)
Mn2_1	16	0.6280(5)	0.125	0.335(3)	1	2.6(3)
O1_1	16	0.9374(16)	0.125	0.355(3)	1	0.9(9)
O1_2	16	0.9374(8)	0.4850(13)	0.6349(19)	1	0.5(4)
O1_3	16	0.9374(8)	0.2351(13)	0.1349(19)	1	0.5(4)
O2_1	16	0.8168(15)	0.125	−0.028(3)	1	1.2(6)
O2_2	16	0.8168(7)	0.2577(14)	0.7060(18)	1	0.5(3)
O2_3	16	0.8168(7)	0.0077(14)	0.2060(18)	1	0.5(3)

Table S18 | Crystallographic data for the nuclear phase of 12R-BPM derived from the Rietveld refinement of the PND pattern collected at 4.2 K. Numbers in parentheses indicate ±1σ. The somewhat large B_{iso} values of approximately 3 for the Mn ions may be an artifact of the limited *Q* range of the data set. The model is consistent with the data. Additional measurements with expanded *Q* ranges may help constrain these values.

References

(1) Hu, Z.; Bertram, S.; Kaindl, G. X-Ray-Absorption Study of PrO_2 at High Pressure. *Phys. Rev. B* **1994**, *49* (1), 39–43. https://doi.org/10.1103/PhysRevB.49.39.

(2) Coelho, A. A. TOPAS and TOPAS-Academic: An Optimization Program Integrating Computer Algebra and Crystallographic Objects Written in C++. *J. Appl. Crystallogr.* **2018**, 51 (1), 210–218.
